# Simultaneous production of biofuel from agricultural wastes and bioremediation of the waste substrates: A review

**DOI:** 10.1016/j.crmicr.2024.100305

**Published:** 2024-11-15

**Authors:** Chukwuemeka Samson Ahamefule, Chidimma Osilo, Blessing C. Ahamefule, Stella N. Madueke, Anene N. Moneke

**Affiliations:** a Department of Microbiology, University of Nigeria, Nsukka 410001, Nigeria; b Department of Applied Microbiology, Faculty of Biosciences, Nnamdi Azikiwe University, Awka, Nigeria; c Department of Microbiology, University of Jos, Jos 930001, Nigeria; d Centre for Environmental Management and Control, University of Nigeria, Enugu campus, Nigeria

**Keywords:** Agricultural waste, Biofuels, Bioremediation, Fermentation, Anaerobic digestion, Zero-waste

## Abstract

Pollution from fossil fuel usage coupled with its unsustainability is currently instigating a global drive for affordable and eco-friendly alternatives. A feasible replacement seems to be microbial biofuels. However, the production cost is still high, partly due to the cost of substrates and media. Microalgae, yeasts, moulds and bacteria can grow on cheap and easily available agricultural waste substrates to produce bioethanol, biogas, biobutanol, acetone and/or lipids for biodiesel. Oleaginous microbes, such as several species of *Chlorella, Schizochytrium, Rhodotorula, Rhodosporidium, Yarrowia, Aspergillus, Rhodococcus,* etc., have been applied in the production of high and choice lipids for biodiesel. High bioethanol, butanol and acetone yields have also been achieved with several agricultural waste substrates either through separate hydrolysis and fermentation or simultaneous saccharification and fermentation. The isolation or transformation of some microbial strains has shown the possibility of using only one microorganism to produce bioethanol from lignocellulose biomass in consolidated bioethanol production. High biogas/biomethane yield has also been recorded from different agricultural wastes, especially in co-digestion systems. Pre-treatment, detoxification, application of microbial co-cultures, co-substrate mixing ratios, organic loading rate, hydraulic retention time among others have been reported to affect the quantity and quality of different biofuels produced from agricultural wastes. Interestingly, these agricultural waste substrates were greatly degraded during fermentation and/or digestion to achieve high level of bioremediation. Therefore, this study presents a holistic review of various agricultural wastes adopted as biofuel substrates, the conditions to attain optimum biofuel productions and cases of simultaneous bioremediation of substrates obtained during biofuel production. The valorization of the biofuel by-products into other essential products to achieve a zero-waste and circular bioeconomy is also properly presented.

## Highlights

• Fossil fuels are unsustainable and greatly contribute to global warming.
• Microbial biofuels could be veritable substitutes to fossil fuels.
• The cost of producing microbial biofuels is still high, partly due to substrates.
• Agricultural waste substrates are cheaper and sustainable alternatives.
• There is simultaneous bioremediation of agricultural waste used as substrates for biofuels.

## Graphical abstract

**Figure:**
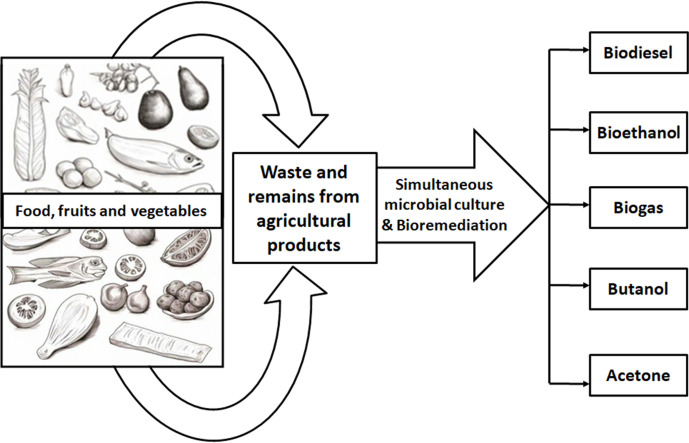

## 1 **Introduction**

Massive pollution from fossil fuel usage and its rapidly depleting status, among other challenges, have increased the quest for sustainable and eco-friendly energy sources. Microbial biofuel is a potential sustainable replacement (Azni et al., 2023; Martins et al., 2019; Perera, 2018). However, the cost of substrates/media used in microbial fuel synthesis contributes greatly to the production cost of these biofuels (Raut et al., 2023). This challenge can be addressed by using cheap and available agricultural wastes as substrates. Agricultural wastes constitute a large proportion of municipal waste, especially with the current increase in global population. These agricultural wastes include waste from cultivating and processing agricultural products like fruits, vegetables, grains, dairy, poultry, meat, crops, etc. (Raut et al., 2023; Nguyen et al., 2018; Louhasakul et al., 2019; Chan et al., 2020). The applications of these wastes for biofuel productions also have the benefit of biodegrading the substrates into other essential by-products (such as biofertilizers, biochar, etc.) which promotes zero-waste and circular bio-economy (Musetsho et al., 2021; Ji et al., 2015; Liu et al., 2017; Jin et al., 2022; Hung et al., 2017).

Oleaginous microalgae, yeasts, moulds and bacteria can accumulate high lipids from either raw or pre-treated agricultural wastes. The lipids are extracted and transesterified to generate biodiesel. Bioethanol, acetone and/or butanol are produced from the biochemical fermentations of sugars by fungi and/or bacteria either as single or co-cultures. Biogas is the product of anaerobic digestion of organic matter. One or more of these biofuels have been reported to be produced sequentially or simultaneously from bagasse and other lignocellulose wastes, food and chicken wastes/remains, several whey, palm oil mill effluents (POME) and other wastewater by-products, animal waste and dung, etc. (Louhasakul et al., 2019; Musetsho et al., 2021; Wang et al., 2020; Koutra et al., 2019; Carota et al., 2020). Interestingly, over 80 % bioremediation has been reported when some of these agricultural waste substrates were used for producing biofuels (De Bhowmick et al., 2019; Abdulsamad and Varghese, 2017; Nurjuwita et al., 2021). This review gives a comprehensive insight into the various agricultural wastes that have been explored in biofuel productions, the microorganisms and culture conditions employed for optimal yields as well as the bioremediation achieved due to fermentation of the substrates, mostly within the last decade (except for a few cases of lipids from moulds). The challenges encountered in valorizing agricultural wastes to biofuels and ways of addressing them, as well as the future prospects of using agricultural wastes as substrates are also elucidated.

## 2 Agricultural wastes as cheap substrates for biofuels

**Fig. 1:**
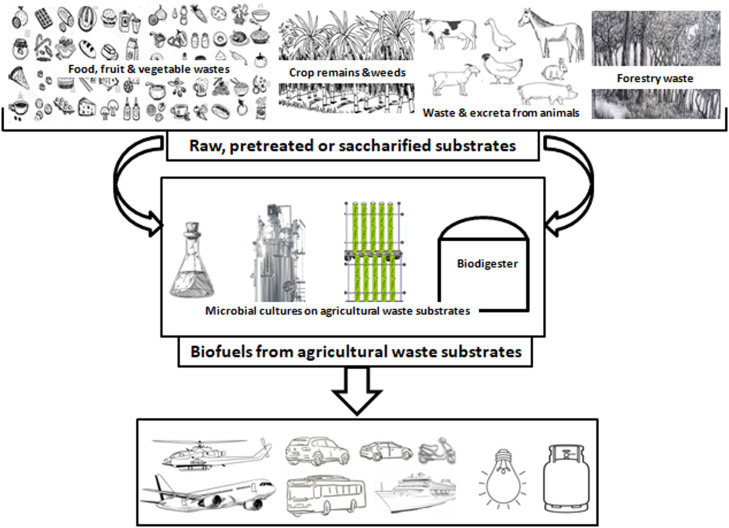
Applications of agricultural waste substrates for biofuel productions.

Agricultural wastes are by-products, remains and spoilt components of raw and processed food, fruits and vegetables. They also include excreta from farm animals, lignocellulose biomass from crops, weeds, trees, etc. Agricultural wastes can generally be categorized into four groups, viz: (i) crop waste, e.g. rice bran and husk, wheat straws, sugarcane bagasse, etc.; (ii) animal waste, e.g. animal dung and manure, whole or parts of dead animals, etc.; (iii) processing waste, e.g. packaging materials, fertilizer cans, etc.; and (iv) hazardous waste, e.g. pesticides, insecticides, etc. The annual global agricultural waste generation is estimated at 998 million tons (Raut et al., 2023). And with the current global population rise as well as efforts to increase food production, agricultural waste is expected to keep increasing in the future (Santeramo, 2021; Daszkiewicz, 2022; Pawlak and Kołodziejczak, 2020). Regrettably, when these wastes are not properly disposed of, they constitute environmental pollution and avenue for the growth and dissemination of pathogens (Siddiqua et al., 2022; Abubakar et al., 2022). These organic agricultural wastes (80 % of agricultural wastes is organic) could be valorized into important biofuels such as biodiesel, bioethanol, biogas, biohydrogen and advanced biofuels like acetone and butanol (Fig. 1) (Raut et al., 2023; Musetsho et al., 2021; Siwina and Leesing, 2021; Sharma and Sharma, 2023; Sumardiono et al., 2023; Valles et al., 2020).

The cost of substrates or media in biofuel production usually account for a significant part of the total production cost, for example, this accounts for about 75 % total cost in biodiesel production (Alrefai et al., 2021). Therefore, the use of waste agricultural substrates as substitutes for conventional media would go a long way toward reducing the cost of production. Several kinds of agricultural wastes have been valorized into different biofuels using bacteria, yeasts, moulds or microalgae (Louhasakul et al., 2019; Dong et al., 2019; Qiao et al., 2018; Behera et al., 2019; Srivastava et al., 2021). Wastes/remains from cooked and processed foods with high amounts of easily biodegradable carbohydrates (i.e. simple sugars and starch) and lipids have been applied as choice substrates in biofuel production without pre-treatment (Wang et al., 2020; Zeng et al., 2018; Johnravindar et al., 2018). Furthermore, the abundant lignocellulose agricultural wastes, from harvested crop remains, weeds, forestry, etc., have also been demonstrated as potential substrates for different biofuel production (Fig. 1) (Carota et al., 2020; Dong et al., 2019; Miao et al., 2020; Cheirsilp and Kitcha, 2015). These lignocellulose substrates are usually pre-treated and hydrolyzed to remove the recalcitrant lignin components and to release biodegradable carbons using physical, chemical and/or biological methods (Siwina and Leesing, 2021; Liu et al., 2020; Shen et al., 2018; Catherine and Twizerimana, 2022; Jiménez et al., 2020). Excreta and other waste from farm animals such as cows, pigs, sheep, poultry, etc., are also being used as either sole- or co-substrates in producing biofuels, especially biogas or microbial lipids for biodiesel (Musetsho et al., 2021; Yan et al., 2018; Gao et al., 2021; Jin and Wei, 2023).

### 2.1 Biodiesel from agricultural waste substrates

Biodiesel from microbial biomass could be a viable and sustainable alternative to fossil diesel, currently a major contributor to environmental pollution and the accumulation of greenhouse gases. Biodiesel is obtained from the transesterification of lipids or fats and oil (Yen et al., 2019; Singh et al., 2020; Selvakumar and Sivashanmugam, 2018; Leesing et al., 2021). Some oleaginous microorganisms can accumulate high amount of choice lipids that are essential for biodiesel production (Nguyen et al., 2018; Qiao et al., 2018; Miao et al., 2020; Kumar et al., 2015). These microbial lipids are mainly composed of triglycerides (TAGs) with fatty acids consisting of oleic (36 – 60 %), palmitic (27 – 45 %), stearic (6 – 14 %), and linoleic (6 – 15 %) acids (Ngamsirisomsakul et al., 2021; Selvakumar and Sivashanmugam, 2017; Nair et al., 2020). The use of agricultural wastes as either sole- or co-substrates has greatly helped to reduce the cost of microbial biodiesel production while also adequately creating avenues for the simultaneous bioremediation of such wastes (Vyas and Chhabra, 2019; Karim et al., 2021).

Some research works have reported the production of lipids from microalgae, yeasts, moulds and bacteria using assorted kinds of agricultural wastes showing the great potentials of using several microbes to valorize waste substrates to lipids/biodiesel (Musetsho et al., 2021; Carota et al., 2020; Behera et al., 2019; Schambach et al., 2020; Hashem et al., 2020). Several species of *Chlorella,* as well as *Schizochytrium* sp., *Crypthecodinium cohnii, Acutodesmus obliquus, Tetradesmus obliquus, Diplosphaera* sp., etc., have been demonstrated to produce lipids from sugarcane bagasse, food waste hydrolysate (FWH), poultry manure, dairy and winery wastewaters, among others, in either heterotrophic or mixotrophic conditions (Table 1). Some of these agricultural waste media or supplemented waste media were shown to even produce more lipids in some microalgae species than the conventional control media. For example, *Chlorella protothecoides, A. obliquus, C. cohnii* and *Monoraphidium* sp. were reported to have produced higher amounts of lipids from sugarcane bagasse hydrolysate, poultry litter extract, exhausted olive pomace and walnut shell extract, respectively, than the various control media used, such as glucose supplemented medium, BG-11 and other synthetic media (Table 1) (Musetsho et al., 2021, Dong et al., 2019; Mu et al., 2015; Paz et al., 2021). Therefore, the commercialization of lipids for biodiesel production using these microalgal strains and waste substrates would not only lead to the production of more lipids (than conventional media) but at a lower production cost.

**Table 1:** Production of lipids from microalgae grown on agricultural wastes.

Waste substrate	Composition/ratio of waste medium	Microalga	Cultivation time (day)	Cultivation conditions	Lipid/biodiesel production	Reference
Sugarcane bagasse hydrolysate (SBH)	SBH was used as a source of reducing sugar in basal medium	*Chlorella protothecoides*	8	28 °C at 160 rpm without light	The lipid productivity obtained from SBH (493 mg/L/d) was about 4.9 and 1.88 fold higher than that from glucose (100.62 mg/L/d) and artificial mixture sugars (262.26 mg/L/d), respectively, by 96 h of cultivation at the highest level of biomass	(Mu et al., 2015)
	Basal medium supplemented with different concentrations of SBH (5 – 40 g/L)	*Schizochytrium* sp.	5	Heterotrophic condition at 25 °C	The 40 g/L SBH medium gave the highest lipid content (45.15 %) compared to similar concentrations of refined glucose medium control and other concentrations of SBH media	(Nguyen et al., 2018)
Food waste hydrolysate (FWH)	Different dilution concentrations (10, 20, 30 and 40 g/L) of reducing sugar in FWH	Single and co-culture of *Chlorella vulgaris* and yeast	5	28 °C, 1.8 klux at 180 rpm	Microalgal lipid production consistently increased with increasing reducing sugar in the co-culture from 1.5 g/L (in 10 g/L FWH) to 3.5 g/L (in 40 g/L FWH). However such an increase was not observed in the microalgae mono-culture.	(Zeng et al., 2018)
	FWH was used to supplement Bold's basal medium as a source of sugar	*Auxenochlorella protothecoides*	5	25 °C at 180 rpm	FWH produced lipid content and yield of 5.64 g/L and 0.216 g g^-1^_sugars_, respectively	(Patel et al., 2019 Dec 1)
	FWH concentrations of 0, 1, 3, 5, 7 and 10 % (v/v) were supplemented with 0, 5, 10, 50, 100 and 200 mM crude glycerol (CG)	Engineered *Phaeodactylum tricornutum* (DUALOE)	9	20 °C, 200 μmol photons/m^2^/s irradiance with light-dark cycle of 12:12 h at 160 rpm	The highest total lipid (134.7 mg/L) and triacylglycerol (120 mg/L) were obtained with 5 % FHW and 100 mM of CG	(Wang et al., 2020)
Poultry litter extract (PLE)	Both raw and acid pre-treated PLE were used as the main media	*Acutodesmus obliquus*	14	25 °C, 80 µmol photons/m^2^/s with light-dark cycle of 16:8 h	More lipid content (27.42 %) and productivity (38.49 mg/L/d) were obtained from pre-treated PLE medium compared to BG-11 control which gave 22.45 % and 27.98 mg/L/d, respectively	(Musetsho et al., 2021)
Exhausted olive pomace (EOP)	Yeast extract supplemented EOP was used as main medium	*Crypthecodinium cohnii*	7	25 °C, dark condition at 160 rpm	Higher total lipid production (1.78 g/L) and lipid productivity (0.25 g/L/d) were obtained from 8 g/L EOP medium compared to that from the best synthetic medium - 1.13 g/L and 0.16 g/L/d, respectively	(Paz et al., 2021)
Walnut shell extract (WSE)	WSE + NaNO_3_ (1.16 g/L) supplemented with various concentration of CO_2_ (4, 8, 12 and 16 %)	*Monoraphidium* sp.	7	25 °C, continuous illumination at 6500 lux	WSE with 12 % CO_2_ gave the highest lipid content and productivity of 49.54 % and 97.52 mg/L, respectively, which were significantly (*P* < 0.01) higher than those obtained from the control medium	(Dong et al., 2019)
Duckweed waste (DWW)	The supernatant of DWW (5, 10, 20 and 40 %) from bio-H_2_ fermentation supplemented with 1 mM ammonium was used	*Chlorella sacchrarophila*	15	30 °C, at 50 μmol/m^2^/s light density, with a light-dark ratio of 12/12-h	There was a significant increase in lipid content and production with increasing DWW concentration compared to 0 % DWW medium. The 20 % DWW medium gave the respective optimum values of 34.4 % and 270 mg/L	(Schambach et al., 2020)
Chicken manure digestate	25 % digestate effluent medium from chicken manure	*Tetradesmus obliquus* co-cultured with actinomycete (*Nocardia bhagyanaraya*)	12	25 °C, 10.8 μmol/m^2^/s continuous illumination	Co-culture with actinomycete gave significantly (*P* < 0.05) higher lipid production of 239.6 mg/L compared to that from microalgal monoculture, 133.5 mg/L	(Kumsiri et al., 2018)
Dairy and winery wastewaters	Dairy (33 %) and winery (50 %) wastewaters used as main media	*Diplosphaera* sp.	14	24 °C, ∼200 µmol/m^2^/s with light-dark cycle of 14:10 h	There were 43.07 and 16.98 % (total solid) lipid production compared to 26.46 % obtained from BG-11 control	(Liu et al., 2016)
Digestate of agro-waste such as expired dairy products, cow-, swine- and chicken-manure, and slaughterhouse wastes	Only digestate and digestate supplemented with other components like glucose, MgSO_4_, trace elements and cheese whey were used	*Parachlorella kessleri*	18	25 °C, 370 µmol photons/m^2^/s at 250 rpm and constant aeration of 0.5 L/min	Final lipid contents obtained from waste media inoculated with autotrophic inoculum were higher than those from mixotrophic inoculum. These include digestate supplemented with cheese whey which gave the highest final lipid concentration of 229.6 mg/L compared to only digestate (121.64 mg/L) and digestate supplemented with (i) 0.5 and 5 g/L glucose (162.58 and 28.13 mg/L); (ii) glucose and trace elements (10.24 mg/L); (iii) glucose and MgSO_4_ (58.97 mg/L)	(Koutra et al., 2019)

The co-culture of microalgae with other microorganisms on agricultural wastes has also been demonstrated to help increase lipid accumulation in such microalgae. The plant-promoting actinomycete, *Nocardia bhagyanarayanae* I-27, was shown to significantly enhance the growth and lipid-producing ability of *Tetradesmus obliquus* in chicken manure digestate. The co-culture resulted to over 75 % lipid increase – from 133.5 mg/L in the microalga mono-culture to 239.6 mg/L in the co-culture (Kumsiri et al., 2018). Also, Zeng et al. (2018) stated that the co-culture of the yeast, *Rhodosporidium toruloides,* helped to both increase lipid productivity of the microalga, *Chlorella vulgaris*, and enhanced the efficiency of bioremediation of the waste substrate, FWH. Similarly, the metabolic engineering of microalgae species is yet another option for enhancing lipid productivity from waste substrates. For instance, the engineered *Phaeodactylum tricornutum* was reported to give the highest lipid and triaceylglycerol production using FWH supplemented with crude glycerol (Table 1) (Wang et al., 2020). Furthermore, the pre-treatment of some agricultural waste substrates has been reported to better increase lipid accumulation in microalgae than the unpretreated substrates. In a study with *A. obliquus*, the microalga accumulated more lipids (27.42 %) when grown with acid-pre-treated poultry litter than when cultured with the raw substrate (22.45 %) (Musetsho et al., 2021).

Some oleaginous yeast strains have been demonstrated to accumulate high lipid contents with some giving over 50 % from different agricultural wastes (Louhasakul et al., 2019; Miao et al., 2020). These include several species of *Rhodotorula* (especially *Rt. toruloides*), *Yarrowia lipolytica, Rhodosporidium toruloides* (*Rs. toruloides*), *Lipomyces starkeyi*, etc. (Table 2). These yeast strains were mostly grown in mesophilic conditions with shaking/agitation in either batch or fed-batch culture conditions. Agricultural wastes such as POME, sugarcane bagasse, wastes from soy molasses and whey, food, fruit and vegetable remains, etc. have been adequately valorized in the production of lipids in these oleaginous yeasts (Table 2) (Ngamsirisomsakul et al., 2021; Karim et al., 2021; Louhasakul et al., 2020; Gao et al., 2020; Wang et al., 2021).

**Table 2:** Production of lipids from yeasts grown on agricultural wastes.

Waste substrate	Composition/ratio of waste medium	Yeast	Cultivation time (h)	Cultivation conditions	Lipid/biodiesel production	Reference
Palm oil mill effluent (POME)	POME with 2 % crude glycerol (CG) and biosurfactant from *Bacillus subtilis*	*Yarrowia lipolytica*	72	30 °C at pH 6.0 and 140 rpm	The addition of biosurfactant resulted to 1.25-fold increase in lipids from 2.04 g/L, in medium without biosurfactant, to 2.54 g/L, in medium with biosurfactant	(Louhasakul et al., 2020)
	Raw (100 %) and diluted (50 %) POME	Single and co-cultures of *Lipomyces starkeyi* with *Bacillus cereus*	144	30 °C at pH 7.0 and 150 rpm	The co-culture gave the highest lipid accumulation of 2.27 g/L compared to the highest obtained in the single culture of the yeast (1.62 g/L) or bacterium (1.46 g/L)	(Karim et al., 2021)
	POME supplemented with 1–8 % (w/v) CG	*Candida tropicalis* and *Y. lipolytica*	72	30 °C at pH 6.0 and 250 rpm	*Y. lipolytica* gave a higher lipid content of 52.7 % than *C. tropicalis* (33.5 %), while their co-culture gave 47.7 % lipid content. The 2 % CG gave the best lipid production	(Louhasakul et al., 2019)
Sugarcane molasses (SM)	Waste extract with 30 g/L SM	*Rhodosporidium* (*Rs.*) *toruloides*	192	Fed-batch fermentation at 30 °C and 120 rpm	Lipid production and productivity of 39.2 g/L and 0.25 g/L/h, from SM-fed medium were higher than that from glucose-fed medium control, 29.4 g/L and 0.19, respectively	(Singh et al., 2020)
Sugarcane bagasse hydrolysate (SBH)	Supplemented SBH medium	*Rs. toruloides, Rhodotorula* (*Rt.*) *glutinis, Trichosporon cutaneum* and *Y. lipolytica*	72	30 °C at an initial pH of 6.45–6.52 and 200 rpm	All the yeast strains gave good lipid accumulation in the SBH medium. *Rt. glutinis* gave the highest lipid production (13.18 g/L) and content (37.8 %) while *Y. lipolytica* gave the least of 7.58 g/L and 30.8 %, respectively	(Ngamsirisomsakul et al., 2021)
	Supplemented SBH medium	*Candida tropicalis*	168	30 °C at an initial pH of 5.5 - 6.0 and 150 rpm	Single cell oil (SCO) content, production, productivity and yield of 26.5 %, 1.54 g/L, 0.22 g/L/d and 0.043 g g^-1^ sugar, respectively, were produced from undetoxified SBH. Furthermore, 10.8 g biodiesel was obtained from SCO from 1000 g raw sugarcane bagasse	(Hassa et al., 2024)
Corn cob	Detoxified corn cob hydrolysate supplemented with 5 g/L yeast extract	*Rt. taiwanensis*	120	26 °C at pH 7.0 and 200 rpm in 5 L fermentor	Production of 60.3 % (w/w) oil based on biomass and 55.8 g/1000 g lipid coefficient corncob	(Miao et al., 2020)
Wheat straw (WS)	Different pre-treated WS hydrolysate (WSH): washed cellulosic hydrolysate (WCH), decolorized cellulosic hydrolysate (DCH), concentrated & decolorized cellulosic hydrolysate (CDCH), post hydrolyzed two-fold concentrated & decolorized hydrolysate (C2DH), and the control, synthetic concentrated medium hydrolysate (SCM)	*Rs. toruloides* and *L. starkeyi*	120	30 °C at pH 5.3 and 250 rpm	*Rs. toruloides* gave the highest lipid concentration of 3.36 g/L and productivity of 34.98 mg/L in DCH and the least in CDCH compared to that of SCM (4.50 g/L and 3.03 mg/L), respectively. While *L. starkeyi* gave the highest lipid concentration and productivity in both DCH and WCH - 12.57, 17.32 g/L and 33.59, 33.22 mg/L - compared to that of SCM (16.75 g/L and 29.27 mg/L), respectively	(Liu et al., 2020)
Waste cooking oil (WCO)	Medium supplemented with 60 g/L CG and WCO (5 – 50 g/L)	*Rt. glutinis*	192	24 °C at pH 5.5	Medium supplemented with 30 g/L WCO gave the highest lipid content (46.5 %) compared to that of the control (39 %) without WCO	(Yen et al., 2019)
Waste sweet potato vines (WSV)	Several pre-treated WSV: autoclaved-treated WSV (ATWSV), acid-hydrolyzed WSV (ACWSV) and alkaline-hydrolyzed WSV (ALWSV)	*Trichosporon fermentans*	288	30 °C at 5.5 initial pH	There was significant lipid accumulation in the ATWSV medium compared to the others at both separate hydrolysis and fermentation (SHF) and simultaneous saccharification and fermentation (SSF) conditions. There was also higher lipid yield and content of 6.98 g/L and 36 % from SSF compared to that from SHF (2.7 g/L and 25 %), respectively	(Shen et al., 2018)
Cheese whey (CW)	Untreated CW (UCW) and deproteinized CW (DCW) at different concentrations - 25, 50, 75 and 100 % - supplemented with some trace elements	*Cystobasidium oligophagum*	168	28 °C at 150 rpm	DCW medium gave higher lipid productivity at all concentrations compared to UCW with the 100 % DCW medium giving 0.0335 and 0.0272 g/L/h, respectively. However, the 25 % medium gave the highest lipid contents of 52.48 and 43.13 % for both DCW and UCW, respectively	(Vyas and Chhabra, 2019)
Food waste (FW), fruit and vegetable waste (FVW) fermentate	Undiluted supernatant from FW and FVW fermentation effluent	*Y. lipolytica*	264	28 °C at 6, 7 & 8 initial pH and 180 rpm	There were higher lipid concentrations of 3.20 and 3.08 g/L, contents of 21.52 and 26.02 % and yields of 0.091 and 0.139 at pH 8 from FW and FVW media, respectively, with <3 h lag phase. While pH 6 gave the least concentrations of 1.35 and 1.19 g/L, contents of 14.78 and 17.58 % and yields of 0.048 and 0.054 from FW and FVW media, respectively, with 2–3 days lag phase	(Gao et al., 2020)
Food waste leachate (FWL)	FWL from three digesters diluted to ∼ 25 g/L carbohydrate content	*Cryptococcus curvatus, Rt. glutinis* and *Y. lipolytica*	144	30 °C at pH 6 and 140 rpm	The three different digestates from FWL gave higher lipids for all the yeasts compared to the synthetic glucose medium. *Y. lipolytica* and *Rt. glutinis* gave the highest lipid contents close to 50 % in FWL from digesters with anaerobic sludge and that without inoculum	(Johnravindar et al., 2018)
Food waste fermentation effluent (FWFE)	Supernatant from anaerobic FWFE diluted to 2.5 g volatile fatty acid (VFA)/L	*Y. lipolytica*	720	28/38 °C at initial pH 6.0/uncontrolled and 180 rpm	Lipid concentration, 0.370 g/L, and productivity, 0.12 g/L/d, obtained from VFA at 28 °C and 6.0 pH were the highest from FWFE and the closest to that of synthetic VFA (0.682 g/L and 0.23 g/L/d, respectively)	(Gao et al., 2017)
Orange peel waste (OPW)	OPW extract supplemented with 0.6 g/L (NH_4_)_2_SO_4_	*Rs. toruloides* and *Cryptococcus laurentii*	96	30 °C at pH 5.5 and 500 rpm in stirred tank reactor	Peak lipid production of 5.47 and 4.46 g/L, and a biodiesel yield of 36.90 and 31.90 % were obtained from *Rs. toruloides* and *C. laurentii,* respectively	(Carota et al., 2020)
Potato wastewater	Potato wastewater supplemented with glycerol to give a glycerol concentration of 30 g/L	*Rt. gracilis*	120	20/28 °C at pH 5.0 and 300 rpm/min	The highest total lipid content was obtained by 96 h of fermentation at both 28 and 20 °C - 16.33 and 19.55 g/100 g DCW, respectively. Culture at 20 °C gave higher lipid yield all through fermentation	(Kot et al., 2020)
Waste cooking oil (WCO) from food waste	Several concentrations of WCO (0–100 %) and glucose (42.2 g/L)	*Rs. toruloides*	168	27 °C at variable pH - 4.0 and 11 for sterilization and cultivation, respectively - and 180 rpm	The 25 % WCO medium gave over 85 % more lipid content (5.65 g/L) above that from the medium with only glucose (3.05 g/L). However, lipid production started dropping as the concentration of WCO increased, with 100 % WCO giving the least lipid content	(Gao et al., 2022)
Soy Molasses (SM) and Whey powder (WP)	Pre-treated SM and WP were used separately as carbon sources in the lipid production (LP) medium	*Aureobasidium namibiae*	120	30 °C at pH 6.0 and 300 rpm in a 10 L fermentor	Both SM and WP gave high lipid productions of 5.30 and 5.23 g/L, respectively	(Wang et al., 2021)
Durian peel waste	Detoxified and undetoxified durian peel waste hydrolysate (DPWH) were used as carbon source in the LP medium	*Rt. mucilaginosa*	168	30 °C at pH 5.5 and 150 rpm	Both detoxified and undetoxified DPWH gave 16 % lipid content higher than that obtained from glucose (15 %) and xylose (13 %) controls. Detoxified and undetoxified DPWH also gave similar lipid yield of 1.68 g/L.	(Siwina and Leesing, 2021)
	Supplemented xylose-rich DPWH	*Candida viswanathii*	120	30 °C at 150 rpm	An SCO content and accumulation of 35.3 % and 5.1 g/L, respectively, from undetoxified DPWH were obtained. Maximum fatty acid methyl ester (FAME) yield of 94.3 % was obtained from the SCO	(Fiala et al., 2024)
*Miscanthus* x *giganteus* (Mxg)	Mxg sugar hydrolysate, MSH (60 – 100 g/L), supplemented with ammonium sulphate and amino acid	Two strains of *Rs. toruloides* - Y-27012 and Y-6987	120	28 °C at pH 6.0 and 800 rpm	Accumulated lipids in the two strains increased with increasing sugar concentration in the MSH medium. Highest lipid concentrations of 11.0 and 10.4 g/L were obtained for Y-6987 and , respectively, in 100 g/L MSH medium	(Banerjee et al., 2024)
Oilcane bagasse	Oilcane bagasse hydrolysate (80 g/L) supplemented with yeast nitrogen base salts and yeast extract	Engineered *Rs. toruloides*	144	30 °C at pH 5.6 in 75 L reactor with 40 L working volume	Highest lipid titer value of 8.8 g/L was obtained at 12 h. A total lipid of 103.4 g was extracted from 352 g of freeze-dried yeast pellet which represents 29.7 % yield	(Naik et al., 2024)

Just as in microalgal lipids, higher lipid contents have also been reported in yeast strains using agricultural wastes compared to the standard conventional/control media. In two different studies, *Rs. toruloides* was reported to produce lipids of 39.2 and 5.65 g/L from sugarcane molasses and waste cooking oil (obtained from food wastes) compared to 29.4 and 3.05 g/L derived from glucose media, respectively (Singh et al., 2020; Gao et al., 2022). In another study, two strains of *Rs. toruloides,* Y-6987 and Y-29,012, were demonstrated to produce higher lipids (11.0 and 10.4 g/L) from *Miscanthus x giganteus* (Mxg) hydrolysate than that obtained from both refined glucose and xylose media controls (Banerjee et al., 2024). Attaining higher lipid yields from yeast strains grown on waste substrates (compared to conventional media), presents a great opportunity of commercializing biodiesel from oleaginous yeasts to become competitive with fossil diesel.

The co-culturing of oleaginous yeasts with either bacteria or some microbial metabolites has been demonstrated to help increase lipid production. Karim et al. (2021) showed that co-culturing *L. starkeyi* with *Bacillus cereus* in POME increased the lipid accumulation to 2.27 g/L as against 1.62 g/L or 1.46 g/L obtained from the single cultures of the yeast or bacterium, respectively. The addition of biosurfactant produced by *Bacillus subtilis* TD4 into the POME culture of *Y. lipolytica* also increased lipid accumulation by 1.25 fold (Louhasakul et al., 2020) (Table 2).

Furthermore, several other techniques have been applied to increase lipid accumulation in yeasts using agricultural wastes. The pre-treatment of some waste substrates have been reported to increase lipid production. Several pre-treatment methods such as thermal, chemical, enzymatic, mechanical, etc., have been used alone or in combinations to achieve higher lipid production in yeasts. Autoclaved pre-treated waste sweet potato vines (WSV) was reported to significantly increase lipid accumulation in *Trichosporon fermentans* compared to other pre-treated WSV (i.e. acid- and alkaline-pretreatments) and the unpretreated sample (Shen et al., 2018). Deproteination and heating have also been demonstrated to increase lipid accumulation by *Cystobasidium oligophagum* and *Aureobasidium namibiae* using cheese whey and soy molasses/whey powder, respectively (Vyas and Chhabra, 2019; Wang et al., 2021). Interestingly, some undetoxified pre-treated lignocellulose substrates have been shown to be efficient nutrient sources for lipid production by some yeast strains just like the detoxified samples. For example, both detoxified and undetoxified durian peel waste were reported to produce similar high lipid in *Rt. mucilaginosa* which were about 16 % greater than that obtained using glucose or xylose media controls (Siwina and Leesing, 2021). While *Candida viswanathii* accumulated 5.1 g/L single cell oil that gave high yield of fatty acid methyl ester (94.3 %) (Fiala et al., 2024). This is indeed a giant leap forward towards commercializing microbial biodiesel from lignocellulose biomass in which much resources are spent on detoxifying pre-treated substrates.

The supplementation of agricultural waste substrates with other nutrient sources is another strategy that has been reported to boost lipid production in yeasts. Waste substrates, such as POME and waste cooking oil, that have very high nitrogen content have been supplemented with crude glycerol (a waste carbon source) to balance C/N ratio and increase lipids accumulation by yeasts (Louhasakul et al., 2019; Yen et al., 2019; Louhasakul et al., 2020). While some other waste substrates with high carbon content like corn cob, orange peel wastes, Mxg hydrolysate and oilcane bagasse were reported to be supplemented with yeast extract, ammonium sulphate, yeast nitrogen base and/or amino acids to facilitate lipid accumulation by several strains of *Rs. toruloides* as well as *Rt. taiwanensis* and *Cryptocococcus laurentii* (Carota et al., 2020; Miao et al., 2020; Banerjee et al., 2024; Naik et al., 2024).

Some moulds have been demonstrated to have lipid producing abilities from agricultural wastes. They have been cultivated mostly using solid state or static fermentation and submerged culture conditions (Table 3). Some of these filamentous fungi include: several species of *Aspergillus, Mucor circinelloides, Mortierella isabellina,* etc. (Cheirsilp and Kitcha, 2015; Hui et al., 2010; Kamoun et al., 2019; Ruan et al., 2012). Most of the lignocellulose agricultural wastes used as substrates by these moulds were pre-treated either chemically (acid or alkaline) or enzymatically to delignify, hydrolyze and enhance the substrates as carbon or nutrient sources for fermentation (Table 3) (Ruan et al., 2012; Dey et al., 2011; Kitcha and Cheirsilp, 2014). These pre-treatments were reported to either give better lipid accumulation in the moulds compared to the synthetic media controls or give something close to the control (Ruan et al., 2012; Dey et al., 2011). For example, the crude enzyme-pre-treated chopped rice straw plus wheat bran substrate was reported to give higher lipid yields of 84.30 and 81.73 mg g^-1^ds from *Colletotricium* sp. and *Alternaria* sp. than the yields (68.20 and 60.32 mg g^-1^ds), respectively, obtained from the unpretreated substrate, (Dey et al., 2011). The fact that some of these waste substrates could give higher lipid yields in some moulds than the conventional synthetic media gives tremendous possibilities of future industrialization of biodiesel from moulds.

**Table 3:** Production of lipids from moulds grown on agricultural wastes.

Waste substrate	Composition/ratio of waste medium	Mould	Cultivation time (h)	Cultivation conditions	Lipid/biodiesel production	Reference
Corn cob	Corn waste liquor from corn cob	*Aspergillus* sp.	48	30 °C at pH 5.0 and 150 rpm	There was lipid productivity of 22.1 % compared to that of 23.3 % obtained with the synthetic Sabourauds dextrose broth medium	(Venkata Subhash and Venkata Mohan, 2011)
Chopped rice straw (CRS) and wheat bran (WB)	Crude enzymatic pre-treated CRS and WB (mixed)	*Colletotrichum* sp. and *Alternaria* sp.	192	Solid state fermentation (SoSF) at 28 °C, neutral pH and 50 - 80 % humidity	The crude enzyme-treated medium gave higher lipid yields of 84.30 and 81.73 mg g^-1^ds for *Colletotrichum* sp. and *Alternaria* sp. compared to that (68.20 and 60.32 mg g^-1^ds) of the untreated substrate medium, respectively	(Dey et al., 2011)
Wheat straw (WS) and addictive substrates	3.6 g WS, 0.4 g addictive substrates(wheat bran, sugarcane bagasse, apple peel, orange peel and banana peel) and mineral salt solutions	*Aspergillus oryzae*	144	SoSF at 30 °C and 50 – 80 % humidity	The highest lipid yield, 37.3 mg g^-1^ds, was obtained with wheat bran addictive compared to 35.65, 33.84, 33.4 and 28.5 mg g^-1^ds obtained from sugarcane bagasse, orange peel, banana peel addictive substrates and only WS without addictive, respectively	(Hui et al., 2010)
Corn stover	Acid or alkaline pre-treated corn stover hydrolysate (CSH)	*Mortierella isabellina*	–	25 °C at a pH of 6.0 and 180 rpm	The acid and alkaline CSH gave lipid content and productivity (acid: 34.4 % and 0.050 g/L/h; alkaline: 29.5 % and 0.027 g/L/h) that were relatively close to that of glucose medium, 44.5 % and 0.067 g/L/h, respectively	(Ruan et al., 2012)
Mulberry branches (MB)	3 g MB and mineral salt solution (6 mL)	*Mucor* *circinelloides*	288	SoSF at 28 °C, 6.5 pH and 70 – 80 % humidity	The highest lipid content and yield of 42.43 mg g^-1^ds and 28.8 %, respectively, were obtained by day 6 (144 h) of fermentation	(Qiao et al., 2018)
Wheat bran (WB) and corn straw (CSr)	7.5 g mixture of WB, CSr and glucose (1:1:2 w/w) plus some mineral nutrients - 0.2 g NH_4_Cl, 0.02 g CuSO_4_∙5H_2_O and 0.02 mg veratryl alcohol	*Phanerochaete chrysosporium*	240	Static solid cultivation at 30 °C, and initial humidity of 60 %	There was a maximum lipid content of 30.5 % from the static solid cultivation by day 8 (192 h) which was about 2.44 times greater than the highest obtained from the submerged culture	(Liu et al., 2019)
Bovine whey	Hydrolyzed whey permeate (HWP)	*M. circinelloides f. lusitanicus*	120	33.6 °C at pH 4.5 and 450 rpm optimum agitation	There was maximum lipid content and yield of 32 % and 3.1 g/L, respectively, at optimized agitation and aeration conditions	(Chan et al., 2020)
Rice hull	Rice hull hydrolyzed with sulfuric acid (0.03, 0.05 or 0.09 M) and supplemented with minerals (7 g/L KH_2_PO_4_, 2 g/L Na_2_HPO_4_, 1.5 g/L MgSO_4_.7H_2_O, 1 g/L CaCl_2_.2H_2_O and 0.08 g/L FeCl_3_.6H_2_O)	*M. isabellina*	240	28 °C at pH 6 – 6.4 and 180 rpm	The 0.09 M H_2_SO_4_ hydrolyzed medium gave the highest lipid/oil accumulation and production of 64.3 % and 3.6 g/L, respectively, while 0.03 M H_2_SO_4_ hydrolyzed medium gave the least - 36 % and 0.96 g/L, respectively.	(Economou et al., 2011)
Different agro-industrial sunstrates	Different concentrations (10 – 40 g/L) of carbon source substrates - soap stock of refined soybean (10 40 g/L), soap stock of refined olive oil (20 g/L), waste cooking oil (10 40 g/L) and wheat bran (20 g/L)	*M. circinelloides*	120	30 °C at pH 5.0 and 150 rpm	Some substrates gave higher lipid contents and yields - soap stock of refined olive oil (44 % and 2.44 g/L); 40 g/L soap stock of refined soybean (46 % and 4.07 g/L) and 20 g/L waste cooking oil (51 % and 2.69 g/L) – compared to that from glucose, 38 % and 1.82 g/L, respectively	(Kamoun et al., 2019)
Watermelon peel waste (WPW)	WPW extract diluted with distilled water	*Syncephalastrum* *racemosum*	336	15 - 30 °C at pH 5.0 – 9.0	WPW medium gave higher lipid accumulation and content of 5.12 g/L and 29.4 % compared to that obtained from the optimized synthetic medium, 4.4 g/L and 28.2 %, respectively	(Hashem et al., 2020)
Palm empty fruit bunch (PEFB) and palm kernel cake (PKC)	1 g of PEFB and PKC supplemented with 1 mL of mineral solution	*Aspergillus tubingensis*	168 and 228 for batch and fed-batch, respectively	SoSF at 28 °C, neutral pH and 65 % humidity	The batch culture gave a maximum lipid of 79.9 mg g^-1^ds, the fed-batch, 86.6 mg g^-1^ds, while the repeated-batch culture (with 50 % replacement) gave the highest lipid yield of 91.9 mg g^-1^ds	(Cheirsilp and Kitcha, 2015)
Palm pressed fiber and palm empty fruit branch	Acid or alkaline pre-treated substrate supplemented with 20 – 30 % PKC	*A. tubingensis*	228	SoSF at 28 °C, neutral pH and 40 – 80 % humidity	High lipid production of 88.5 mg g^-1^ds at optimum conditions	(Kitcha and Cheirsilp, 2014)

The supplementation of most of these agricultural wastes with several kinds of synthetic mineral salt solutions is worthy of note (Table 3). This has greatly helped to improve the waste substrates and encouraged better lipid accumulation by the filamentous fungi (Qiao et al., 2018; Cheirsilp and Kitcha, 2015; Hui et al., 2010; Liu et al., 2019). In some cases, agricultural waste substrates were supplemented with other waste substrates for better lipid yields. The pre-treated palm pressed fiber and palm empty fruit branch substrate was reported to be enriched with palm kernel cake in the production of lipid by *Aspergillus tubingensis* (Kitcha and Cheirsilp, 2014).

A few bacterial species have been demonstrated to be capable of accumulating and producing lipids from agricultural wastes. Both wild type (parent) and engineered *Rhodococcus opacus* are the most reported species as well as *Bacillus subtilis* (Table 4) (Kumar et al., 2015; Zhang et al., 2014; Kurosawa et al., 2013; Wells et al., 2015). Behera et al. (2019) reported a unique bacillus species (DS-7), isolated from dairy effluent scum, that could accumulate up to 90 % lipid from lactose medium. This bacterium could also accumulate 72 % lipid from dairy wastewater (DWW), and 84.93 or 83.33 % lipid from DWW supplemented with minimum salt or glucose, respectively. Other agricultural wastes that have been demonstrated as substrates for producing lipids in bacteria are cotton stalk, pre-treated pine effluent, kraft hardwood pulp, cork wastes, etc. (Table 4).

**Table 4:** Production of lipids from bacteria grown on agricultural wastes.

Waste substrate	Composition/ratio of waste medium	Bacteria	Cultivation time (h)	Cultivation conditions	Lipid/biodiesel production	Reference
Cotton stalk	Cotton stalk hydrolysate (1 L) supplemented with 0.5 g/L KH_2_PO_4_ and 1 g/L MgSO_4_·7H_2_O	*Bacillus subtilis*	96	Optimum conditions: 30 °C at pH 7.0, initial sugar concentration of 50 g/L, C/N ratio of 50 in 10 L bioreactor	Maximum lipid productivity and content of 2.3 g/L and 39.8 %, respectively, were obtained at 48 h	(Zhang et al., 2014)
Pine organosolv pretreatment effluent (POPE)	0.5 to 1.5 w/v solids of POPE used as the sole carbon source	*Rhodococcus opacus*	120	Neutral pH	The POPE medium of 1.5 w/v solids gave a maximum lipid content of 26.99 % of bacterial cellular dry weight	(Wells et al., 2015)
Kraft lignin (KL)	Minimal medium with oxygen-treated KL as carbon source	*R. opacus*	120	30 °C at 150 rpm	Maximum lipid of 0.067 mg/mL was obtained at 36 h of fermentation	(Wei et al., 2015)
Unbleached kraft hardwood pulp (UKHP)	Enzyme-pretreated UKHP hydrolysate	Xsp8 - engineered *R. opacus*	168	pH 5	A maximum fatty acid yield of 11.0 g/L corresponding to 45.8 % of dry cell weight was produced.	(Kurosawa et al., 2013)
Dairy wastewater (DWW)	Raw DWW; DWW supplemented with: (a) glucose, (b) minimal salt medium (MSM) in different ratios - 2:1, 1:1 and 1:2	Isolate DS-7	96 h	30 °C at 150 rpm	Maximum lipid content and productivity of 84.93 % and 0.96 g/L/d were obtained from DWW supplemented with MSM in a ratio of 1:1 (even above that supplemented with glucose) while DWW without supplementation gave 72 % and 0.727 g/L/d, respectively	(Behera et al., 2019)
	Raw DWW and that supplemented with 1 % dextrose or MSM (in ratios: 1:1, 1:2 or 1:3)	*R. opacus*	96	30 °C at initial pH of 7.0 and 250 rpm. Bioreactor experiments had both controlled and uncontrolled temperature and pH	Highest lipid production and content of 1.89 g/L and 51–52 %, respectively, were obtained in the controlled bioreactor set-up	(Kumar et al., 2015)
Hydrocarbon-contaminated cork waste	MS medium supplemented with 1 g/L hexadecane (hydrocarbon waste) as a carbon source	*R. opacus*	48	30 °C at 150 rpm in 250 mL flask	There was production of 0.59 g of triacylglycerol lipid (TAG) /g of hexadecane consumed with 0.60 g g^-1^ of TAG content of cellular dry weight (CDW) and 0.54 g TAG/g of hexadecane consumed with TAG content 0f 0.77 g g^-1^ (CDW) in natural and regenerated cork sorbents, respectively	(Castro et al., 2017)

Just like lipid accumulation in fungi, supplementation of agricultural waste substrates has been reported to also increase lipid accumulation in bacteria (Behera et al., 2019; Kumar et al., 2015; Zhang et al., 2014; Castro et al., 2017). Glucose/dextrose, minimal salt medium, magnesium sulphate, monopotassium phosphate, etc. are some supplements that have been used with agricultural waste substrates in bacterial lipid production (Table 4).

Comparatively, lipid production from yeasts and bacteria takes a lesser time on the average, than from microalgae and moulds while using some agricultural waste substrates (Tables 1 to 4). This is understandable as bacteria and yeasts usually have faster growth rates with short generation time. This therefore might make them better candidates in larger-scale lipid production. However, published research works seem to have evaluated and characterize more of oleaginous yeasts that can metabolize several agricultural waste substrates, hence, the reason for more yeasts applications in large-scale lipids production from waste substrates (Table 2). Notwithstanding, some microalgae also have fast growth rate, especially when cultivated in either heterotrophic or mixotrophic conditions. Therefore, some of these algal strains have been reported in large-scale lipid production as well. However, the addition of photo regimen in most microalgal lipid production and the recalcitrant cell wall of some species further make algal lipid production cumbersome and more costly.

Furthermore, several studies have reported single oleaginous fungal or bacterial strains that could directly metabolize complex agricultural waste substrates without external pre-treatment which is very rare among oleaginous microalgae (Kumar et al., 2015; Vyas and Chhabra, 2019; Dey et al., 2011). The ability of some yeast strains to grow and accumulate lipids in undetoxified pre-treated agricultural waste substrates is yet another advantage of using yeast strains for lipid production over other microorganisms (Siwina and Leesing, 2021; Fiala et al., 2024). All these tend to impact on the production cost and have led to the use of such microorganisms in consolidated lipid production.

### 2.2 Bioethanol from agricultural waste substrates

Bioethanol, another important biofuel, can be produced from the biochemical fermentation of simple sugars - generated from agricultural waste substrates - mostly by *Saccharomyces cerevisiae* or *Pichia* sp. (Sharma and Sharma, 2023; Srivastava et al., 2021; Yan et al., 2018; Mihajlovski et al., 2021). To obtain fermentable sugars from agricultural wastes (some of which are lignocellulose biomass), these substrates are usually pre-treated and/or hydrolyzed. Microbial or enzymatic (crude enzymes produced from microorganisms) hydrolyses are usually cheap and effective options adopted in bioethanol production in either separate hydrolysis and fermentation (SHF) or simultaneous saccharification and fermentation (SSF) (Table 5) (Sharma and Sharma, 2023; Mihajlovski et al., 2021; Ire et al., 2016; Sanjivkumar et al., 2018). However, there are cases where commercial enzymes are also employed in hydrolysis/saccharification (Yan et al., 2018).

**Table 5:** Bioethanol production from agricultural wastes.

Agricultural waste	Microorganism/enzymes	Cultivation conditions	Production of reducing sugar	Bioethanol production	Reference
Wheat straw	Hydrolysis: SNH-1 consortium consisting of *Bacillus safensis, Bacillus subtilis, Bacillus circulans, Citrobacter braakii* and *Paenibacillus dendritiformis*; Fermentation: *Saccharomyces cerevisiae*	Hydrolysis optimum condition (from response surface model, RSM): 40 °C at pH 7 and 2 % substrate concentration for 96 h; Fermentation: 30 °C at 120 rpm for 144 h	SNH-1 gave a maximum reducing sugar of 138 mg g^-1^ (27 g/L) from wheat straw hydrolysate (WSH)	WSH gave maximum ethanol of 6.2 % which was close to what was obtained from the 30 g/L glucose control (8.3 %)	(Srivastava et al., 2021)
Several lignocellulosic wastes including: wheat bran, barley bran, rye bran and horsetail waste	Hydrolysis: *Streptomyces fulvissimus*; Fermentation: *S. cerevisiae*	Hydrolysis: Using SoSF, *S. fulvissimus* produced several lignocellulosic enzymes at 30 °C, 120 rpm for 48 h; Fermentation: Reducing sugar was fermented at 30 °C, 120 rpm for 48 h	Crude enzyme produced 1.89, 2.55, 1.81, 1.87 and 2.26 mg/mL reducing sugar from corn stover, horsetail, yellow gentian, cotton fabrics and cotton fabric pre-treated with corona, respectively	Ethanol concentration of 0.46 and 0.39 % were obtained from the highest reducing sugar samples – horsetail and cotton material pretreated with corona, respectively	(Mihajlovski et al., 2021)
Several agro-forestry wastes such as rice straw, wheat straw, rice husk, corn cobs, maize stems, pine wood, pine needle and eucalyptus wood	Hydrolysis: Enzymes from *Bacillus stratosphericus* and *Bacillus altitudinus*; Fermentation: *S. cerevisiae* and *Pichia stiptis*	Hydrolysis: the hydrolytic enzymes produced were used for saccharification of agro-forestry waste at 45 °C, 120 x g for 72 h; Fermentation: RSM optimum conditions of 25 °C at 5.5 pH and 10 % inoculum for 72 h	Maximum reducing sugar of 22.53 mg g^-1^ was produced from organosolv pretreated samples	Highest ethanol concentration and production of 3.72 % and 29.388 g/L, respectively, were obtained from organosolv pretreated samples	(Sharma and Sharma, 2023)
Sugarcane bagasse (SCB)	*Bacillus cereus* and *Bacillus thuringiensis* serovar kurstaki	Optimum conditions for the simultaneous saccharification and co-fermentation were 40 °C, 7.0 pH, and 4 % substrate and inoculum concentrations	–	Maximum ethanol concentration of 19.08 g/L was obtained in the co-culture system with *B. cereus* and *B. thuringiensis*	(Ire et al., 2016)
SCB	*Streptomyces coelicolor*	Aerobic submerged batch fermentation at 30 °C, 7.2 pH and 150 rpm for 21 days	_	Maximum ethanol production of 43.08 g/L was obtained in day 21	(Buraimoh et al., 2021)
Wheat bran (WB), orange peel (OP) and brewer's spent grain (BSG)	*Trametes hirsuta*	Aerobic fermentation at 28 °C and 120 rpm for 30 days	The highest glucose of 7.5 g/L/11day was obtained from OP followed by 6.5 g/L/11day and 6.0 g/L/7 day from BSG and WB, respectively	WB gave the highest ethanol concentration of 1.4 g/L/7day followed by OP (1.0 g/L/11day)	(Carrillo-Nieves et al., 2022)
Cow manure (CM) and anaerobically digested cow manure (ADCM)	Hydrolysis: C1–9 complete enzyme & commercial cellulases SN-1 from *Penicillium oxalicum;* Fermntation: recombinant *S. cerevisiae*	Simultaneous saccharification and fermentation (SSF) at 30 °C and 200 rpm for 72 h	C1–9 gave 25.35 and 15.82 g/L glucose, SN-1 gave 15.87 and 11.60 g/L glucose while both C1–9 plus SN-1 gave 21.05 and 14.66 g/L glucose from treated CM and ADCM, respectively, in batch fermentation	The C1–9 sample gave ethanol concentrations of 18.94 and 12.57 g/L ethanol, the SN-1 sample gave 13.43 and 9.37 g/L ethanol while the C1–9 plus SN-1 sample gave 16.33 and 12.65 g/L ethanol from co-fermentation of glucose and xylose in treated CM and ADCM, respectively, in batch fermentation. However, the highest ethanol concentration of 25.65 and 16.54 g/L were obtained from CM and ADCM, respectively, in fed-batch systems	(Yan et al., 2018)
Several agro-wastes: vegetable residue (VR), mango residue (MR), banana residue (BR), SCB, and sugarcane juice (SCJ)	*Streptomyces variabilis* for saccharification and fermentation	Hydrolysis: saccharification at 30 °C, pH 7.0 and 150 rpm for 48 h; Fermentation: At 28 °C, pH 6.5 and 150 rpm for 5–6 days	*S. variabilis* produced maximum reducing sugar of 59.49, 49.83, 41.44, 40.51 and 35.36 % from 200 IU xylanase pre-treated SCJ, SCB, VR, BR and MR, respectively	Maximum ethanol of 4.69, 2.43, 2.40, 1.94 and 1.63 g/L were obtained from reducing sugar obtained from 200 IU xylanase pre-treated SCJ, SCB, VR, BR and MR, respectively	(Sanjivkumar et al., 2018)
Wasted rice	Hydrolysis: **α**-amylase and glucoamylase; Fermentation: *Pichia kudriavzevii* or *Kluyveromyces marxianus*	Fermentation of thermo chemical-enzymatic pre-treated wasted rice sample at 35 °C, 150 rpm for 48 h	Highest glucose concentration of 430.6 g kg^-1^ wasted rice was obtained	Highest bioethanol concentrations of 15.62 and 15.44 g/L were produced by *P. kudriavzevii* and *K. marxianus*, respectively	(Al et al., 2024)
Cocoa waste	Hydrolysis: cellulase; Fermentation: *S. cerevisiae*	Enzymatic hydrolysis: 150 rpm for 48 h; Fermentation: 32 °C for 72 h	Highest glucose concentration of 10.5 × 10^6^ µg/L was obtained	Purified bioethanol value of 2 mL was obtained with 0.02 % yield from 50 mL cocoa waste suspension	(Nguemfo et al., 2024)
Sugarcane molasses (SCM)	*S. cerevisiae* and *Wickerhamomyces anomalus*	Fermentation of acid pre-treated SCM at 30 °C, pH 4.6, 150 rpm for 72 h	Highest reducing sugar of 53.45 g/L was produced	The monocultures, *S. cerevisiae* and *W. anomalus*, produced maximum ethanol of14.5 and 17.2 g/L, respectively, by 48 h While the co-culture gave 22.24 g/L ethanol by 36 h	(Hawaz et al., 2024)
Rice straw (RS)	Hydrolysis: cellulose hydrolysis of raw RS and different pre-treated RS - dilute sulfuric acid pre-treated (SAP), liquid water pre-treated (LWP) and ferric chloric pre-treated (FCP); Fermentation: *S. cerevisiae*	Enzymatic hydrolysis at 50 °C, 150 rpm for 72 h; Fermentation: at 30 °C and 150 rpm	The glucose yield from raw-RS, LWP, SAP and FCP were 27.9, 32.3, 44.4 and 78.9 %, respectively	The ethanol productions from glucose obtained from raw-RS, LWP, SAP and FCP were 2.01, 2.49, 3.41 and 5.01 g/L, respectively	(Manna et al., 2024)
Spoilage dates	*S. cerevisiae*	Fermentation at 30 °C and 200 rpm	–	Bioethanol value of 47.95 g/L was obtained from spoiled date syrup which was higher than 47 g/L obtained from pure glucose carbon source	(Madi et al., 2024)
Sorghum leaves (SL)	Hydrolysis: cellulase; Fermentation: *S. cerevisiae*	SSF at 35 °C and 120 rpm for 24 h	–	Highest ethanol concentration and productivity of 12.16 g/L and 1.01 g/L/h were obtained	(Naidoo et al., 2024)
*Parthenium hysterophorus* (weed)	Hydrolysis: FPase from *Penicillium citrinum*; Fermentation: *S. cerevisiae*	Fermentation at 30 °C, pH 6.0 and 120 rpm	Highest reducing sugar yield of 0.37 g g^-1^ was obtained within 96 h	Ethanol yield of 0.45 g g^-1^ was produced after 48 h	(Kumar and Aggarwal, 2024)
Oilcane juice	*S. cerevisiae*	Fermentation at 30 °C, pH 5.6 and 300 rpm in 3 L fermentor	–	Bioethanol of 106 g/L was obtained	(Naik et al., 2024)
*Lolium perenne* (ryegrass)	*S. cerevisiae*	Fermentation at 32 °C and 120 rpm	–	Bioethanol concentration of 11 g/L was produced in 72 h with 0.38 g g^-1^ yield from ryegrass press-juice	(Varriale et al., 2024)
Vine shoots	*S. cerevisiae*: SSF of oraganosolve-pre-treated solid hydrolyzed by Cellic® CTec2; *Escherichia coli*: co-fermentation of acid liquor after pre-treatment	SSF at 40 °C, 180 rpm for 72 h; Co-fermentation at 37 °C, pH 6.7, 350 rpm for 72 h	–	Maximum ethanol of 34.0 and 17.6 g/L were produced via SSF at 20 and 10 % substrate loading, respectively. Maximum ethanol of 17 and 19 g/L were obtained after pre-treatment of acid liquor by NH_4_OH and resins, respectively	(Cardoza et al., 2024)
Banana pseudostem sap (BPS)	Hydrolysis: Enzymes (hemicelluloses and cellulose); Fermentation: *S. cerevisiae* and *Pichia stipules*	Hydrolysis at 45 °C, pH 4.5 and 150 rpm; Fermentation at 30 °C at pH 5.0 and 150 rpm for 72 h	Total reducing sugar of 35.52 g/L was obtained from acid-pretreated BPS	Using detoxified acid hydrolysate of BPS, ethanol concentrations of 8.94 and 9.54 mL/L were produced by *S. cerevisiae* from media A (supplemented with malt extract) and B (supplemented with yeast extract), respectively, while *P. stipules* produced 8.07 and 8.58 mL/L ethanol, respectively	(Kumar et al., 2024)

High simple sugar and ethanol (˃ 40 %) have been reported in cases of consolidated bioethanol production (Sanjivkumar et al., 2018; Buraimoh et al., 2021). Some of the bacteria and fungi that have been used separately in consolidated bioethanol production include: *Streptomyces coelicolor, Clostridium thermocellum, Streptomyces variabilis, Trametes hirsute,* etc. (Table 5) (Sanjivkumar et al., 2018; Buraimoh et al., 2021; Nisha et al., 2017; Carrillo-Nieves et al., 2022). These microbes produced ethanol from agricultural wastes, such as sugarcane bagasse, wheat bran, orange peel, rice husk, banana pseudostem, as well as several fruit and vegetable residues either by sequentially or simultaneously hydrolyzing the substrates, and then fermenting the generated reducing sugars to bioethanol (Table 5). This is indeed a great step towards cutting down the cost of bioethanol production, especially when consolidated bioethanol production is designed into an integrated biorefinery (Ahamefule et al., 2024).

Aside pre-treatment, a number of techniques have been adopted to increase both simple sugar and bioethanol production from agricultural wastes. The use of co-cultures in hydrolysis or fermentation has been reported to increase the yields of reducing sugar or bioethanol, respectively, compared to using only monocultures (Srivastava et al., 2021; Ire et al., 2016; Hawaz et al., 2024). The application of the co-culture, *S. cerevisiae* TA2 and *Wickerhamomyces anomalus* HCJ2F, was demonstrated to increase ethanol production from sugarcane molasses (SCM). Bioethanol increased to 22.24 g/L (using the co-culture) from 14.5 or 17.2 g/L when using either *S. cerevisiae* or *W. anomalus*, respectively. Both yeasts also gave very high reducing sugar (53.45 g/L) during hydrolysis of the SCM substrate (Hawaz et al., 2024). *Bacillus cereus* GBPS9 and *Bacillus thuringiensis* serovar kurstaki HD1 co-culture has also been reported to increase ethanol production to a maximum concentration of 19.08 g/L from sugarcane bagasse (SCB) (Ire et al., 2016). Similarly, microbial consortia or enzymes harvested from several co-cultured microbes have been applied for better hydrolysis/saccharification of agricultural waste substrates to increase simple sugar yield needed for fermentation (Sharma and Sharma, 2023; Srivastava et al., 2021). This is very essential in obtaining high yield of fermentable sugars since ethanol output is generally determined by the amount of available sugars.

The immobilization of either microorganisms or enzymes in the saccharification and fermentation of agricultural wastes has also been reported to boost reducing sugar and ethanol yields (Cherian et al., 2015; Sunethra and Rajakumar 2023; Faustine and Djamaan, 2021). Other strategies that have been applied to boost bioethanol production from agricultural waste substrates include: adequate optimization of fermentation conditions (Sharma and Sharma, 2023; Srivastava et al., 2021) including substrate loading rate (especially in fed-batch or continuous/semi-continuous fermentation systems) (Cardoza et al., 2024), detoxification of pre-treated lignocellulose substrates before fermentation (Kumar et al., 2024), etc.

### 2.3 Biogas from agricultural waste substrates

Biogas is a sustainable green energy source that is produced from the anaerobic digestion (AD) of organic matter (Sumardiono et al., 2023; Gao et al., 2021). It is made up of two main constituents, methane (50 – 80 %) and carbon dioxide (30 – 50 %), with little amount of other gases like hydrogen sulphide, hydrogen, nitrogen, carbon monoxide, etc. (Jeung et al., 2019; Usa Onthong, 2017). The process of AD to produce biogas goes through four major sequential stages - hydrolysis, acidogenesis, acetogenesis and methanogenesis (Catherine and Twizerimana, 2022; Jeung et al., 2019). Biogas can be generated from different kinds of agricultural wastes ranging from plant lignocellulose biomass to animal excreta. The AD of these wastes simultaneously leads to their bioremediation to produce biofertilizers and other products (Hung et al., 2017; Gao et al., 2021; Jeung et al., 2019).

Agricultural wastes such as food, fruit and vegetable remains; leaves from trees and different bagasse; straws and husks from rice, wheat, barley, soybeans, maize and rapeseed; spent mushroom substrates and coffee pulps; cow, poultry, pig manure, etc., can all be utilized for biogas production either as mono- or co-substrates (Table 6). Several research works have demonstrated that the co-digestion (coD) of two or more substrates tend to yield better biogas/biomethane than that of single substrates (Potdukhe et al., 2021; Hortence et al., 2023; Muhammad and Chandra, 2021; Poulsen and Adelard, 2016). It was reported that the coD of wheat straw plus activated waste sludge (two substrates) or cow dung plus vegetable food waste plus grass clippings (three substrates) gave higher biomethane of 223.5 mL/g VS or 715 L kg VS^-1^ compared to 36.13 mL/g VS or 257 L kg VS^-1^ obtained from the digestion of wheat straw or cow dung plus grass clippings, respectively (Table 6) (Potdukhe et al., 2021; Poulsen and Adelard, 2016). Other advantages of adopting coD in biogas production from agricultural wastes include: its potential to reduce the generation of hydrogen sulphide and other inhibitory substances (Muhammad and Chandra, 2021; Mudzanani et al., 2021; Edunjobi et al., 2023); it helps to make-up/balance C/N ratios and provides other essential nutrients/minerals, etc. (Edunjobi et al., 2023; Wright, 2021; Xu et al., 2017).

**Table 6:** Biogas production from agricultural wastes.

Agricultural waste	Co-substrate	Source of inoculum	Reactor and digestion condition	Biogas/methane production	References
Date seeds (DS)	–	Sludge - 25, 37.5 and 50 %	Flasks of 500 mL with 400 mL working volume operated for 21 days. The optimum temperature and sludge concentration were 37 °C and 50 %, respectively	Highest biogas or methane/g-VS of 4140 and 3534 or 1143.8 and 949.6 cc/g-VS were obtained from DS at 4.2 or 1.1 g-VS before and after extraction of oil, respectively	(Alrefai et al., 2021)
Spent mushroom substrate (SMS)	Dairy, pig manure and chicken manure	Digested sludge from a straw reactor	Reactor of 600 mL capacity with 350 effective volume, operated at 35 °C and pH 7.0. Inoculum ratio of 30 %, total solid (TS) of 5, 10 & 15 %, and SMS/manure ratios of 1:1, 1:2 & 2:1 were tested	Highest biomethane yields of 114.9 and 105.9 mL/g VS were obtained from SMS plus chicken manure at TS of 15 and 10 %, respectively	(Gao et al., 2021)
Coffee pulp	Chicken feathers	Rumen	1 L digester operated at 30 °C and pH 7.0 for 90 days. Different TS (10, 15, 20 and 25 %) and C/N ratios (25 and 30) were tested	Highest biogas of 10,438.04 mL was produced at 25 (g g^-1^) C/N ratio and 25 % TS with delignified substrate	(Sumardiono and Jos, 2021)
Sweet potato root waste (SPRW)	–	Active inoculum	250 mL capacity conical flask reactor with 150 mL working volume operated at 37 °C. Optimum pre-treatment conditions for biogas were: 2.9 g/L NaOH at 82 °C for 102 min	Highest biogas of 37.8 mL/g SPRW was generated from pre-treated SPRW compared to 28.23 mL/g SPRW from the unpretreated substrate. There was also a 22 % increase in methane (42 to 64 %) at the optimum conditions	(Catherine and Twizerimana, 2022)
Wheat straw (WS), rice straw (RS) and soybean straw (SbS)	Waste activated sludge (WAS)	Rumen	150 mL reactors (BMP bottles) with 100 mL working volume	Co-digestion (coD) of WAS with WS or RS or SbS generated higher cumulative specific biogas/methane of 427/223.5, 388/219 and 340/197.3 mL/g VS compared to 527/36.13,738/88.3 and 314/23.5 mL/g VS obtained from mono-digestion of WS, RS or SbS, respectively	(Potdukhe et al., 2021)
Fish waste	Water hyacinth	Inoculum from biogas plant	Optimum conditions: water hyacinth: fish waste ratio of 25:75 g/250 mL, inoculum of 15 g/250 mL with 95 mL dilution at 37 °C	Maximum biogas having the highest CH_4_ content of 68.17 % was produced in the coD compared to methane contents of 50.22 or 56.68 % from the mono-digestion of fish waste or water hyacinth, respectively	(Hortence et al., 2023)
Sugarcane bagasse (SCB)	-	Cow rumen fluid	2 L digester operated for 60 days. C/N ratios of 25 and 30; biological (with or without 5 % microbial consortium) and physical (ground or raw SCB) pre-treatments were tested	There was higher biogas of 86.85 mL/g TS from ground SCB compared to the raw SCB, 79.9 mL/g TS, at 25 C/N. There was also higher biogas of 88.67 mL/g TS produced in biological pre-treated SCB compared to the unpretreated substrate (49.12 mL/g TS)	(Sumardiono et al., 2023)
Highland barley straw (HBS)	Cow dung (CD) and pig manure (PM)	Anaerobic sequence bioreactor fed with CD	500 mL serum bottles with 350 working volume operated at 30 °C. Substrates were pre-treated with *Trametes* sp. which produced multiple enzymes	Highest cumulative methane of 111.51 mL/g VS was generated from pre-treated substrates (HLB+CD+PM) compared to 68.07 mL/g VS from unpretreated substrates	(Jin and Wei, 2023)
Rice straw (RS)	CD	Bio-digested slurry from CD biogas plant	2 L digester bottles operated at 29–32 °C. RS was pre-treated with microbial consortium containing bacteria and fungi	Highest biogas production of 187.45 L/kg RS was generated from pre-treated RS compared to 128.45 L/kg RS from the unpretreated substrate	(Sahil et al., 2023)
Wheat straw (WS), RS and SCB	–	Effluent from cow manure biogas plant	Glass bottle reactors of 300 mL with 210 mL working volume operated at 35 °C and pH 7–7.5. Substrate to inoculum ratios (S/I) of 1.5 and 2.5 were tested	Highest and least biomethane yields of 339.00 and 15.74 NmLCH_4_/gVS were produced from coD of WS+RS and SCB+RS at S/I of 1.5 and 2.5, respectively	(Meraj et al., 2021)
WS	Untreated wastewater	From AD master reactor	AD was integrated into bio-electrolysis. The bioelectrochemical digester had a 2.3 L capacity with 2.0 L working volume and was operated at 20 mV, 40 mV, 80 mV or 120 mV	An enhanced biomethane yield of 175.17 mL/g COD was generated at 40 mV compared to 105.36 mL/g COD produced by the control without a voltage supply	(Prajapati and Singh, 2020)
Leaf litter of neem (LLN)	Vegetable waste (VW) - composed of beetroot, mustard green and carrot	CD	12 L digester operated at 38 - 40 °C and TS of 8 – 10 % for 30 days. There were coD - LLN+VW (AD1) or VW+CD (AD2) - and mono-digestion - VM (AD3) or LLN (AD) - set-ups	The coD set-ups gave better biogas/methane yield than that of the mono-digestion. The addition of LLN increased biogas and biomethane yields by 87.27 and 91.47 % while reducing H_2_S by 146.73 % compared to AD3. Between the coD, AD2 gave higher methane yield of 0.368 Nm/kg compared to 0.114 Nm/kg from AD1	(Muhammad and Chandra, 2021)
Agricultural by-products, AbP (fruits & vegetables)	Filtered swine manure (FSM); carcasses (CC)	Planting sludge	300 mL Duran bottles operated at 35 °C and pH 7.0. Two phase of experiment: (a) E1- AbP from different seasons and ratios of fruit to vegetables (winter 9:1, autumn 5:5, summer 1:9 & spring 3:7); (b) E2 – best biogas yielding season plus FSM and CC at different mixing ratios	Highest biogas production of 103.3 mL was generated from winter samples followed by 71.5 mL from that of autumn. High biogas, 16.2 mL, and methane yield, 0.84 m^3^/kg VS, were obtained from Mix 1(with FSM, CC and AbP ratio of 3:3:3). The control with only FSM gave the highest biogas of 17.2 mL	(Jeung et al., 2019)
Soybean residues (SbR), papaya peels (PR), SCB, RS, greater galangal (GG)	–	Raw CD plus distilled water in a ratio of 1:1	(a) Batch digester: 500 mL digester flasks and 300 mL storage flask for gas, operated at 34–37 °C and five HRT - 15, 20, 25, 30 and 35 days. (b) Continuous digester: 200 L and 150 L digester and gas storage tanks, respectively, operated at ambient temperature/pressure and at the optimum HRT (from the batch process) for 60 days	For the batch set-up, the highest biogas yields of 560.47 and 404.24 mL were generated from SbR and PP at 25 and 15 days HRT, respectively, while RS and GG gave the lowest biogas yields. The continuous set-up produced average biogas rates of 63.01, 54.63, 16.28, 13.94 and 0.68 L/day from SbR, PP, SCB, RS and GG, respectively	(Usa Onthong, 2017)
Vegetal waste (leaves litter from chestnut, poplar, maple and walnut trees); cheese whey	Thickened sewage-activated sludge, calcium phosphate sediment	–	Glass reactor of 3 dm^3^ capacity with a working volume of 2400 cm^3^ operated at 293, 306 or 326 K and pH 7.4 with a constant agitation of 60 rpm	Maximum biogas/methane yields of 0.72/0.51, 0.68/0.48 and 0.49/0.35 Nm^3^/kg were generated at 326, 306 and 293 K, respectively	(Ivanchenko and Yelatontsev, 2024)
Vegetable food waste (VFW) & grass clippings (GC)	CD, chicken manure (CM) & pig manure (PM)	Digestate from full-scale reactor treated with PM and straw	250 mL serum bottles operated at 55 °C for 70 days	Highest biogas/biomethane was produced from the co-digestion of three substrates compared to two or one substrates. Maximum ultimate biomethane of 697 and 715 L kg VS^-1^ were produced from CD+CM+VFW and VFM+CD+GC with CH_4_/CO_2_ of 2.54 and 3.9, respectively	(Poulsen and Adelard, 2016)
Rapeseed straw	-	Sludge	500 mL reactors with 200 mL working volume operated at 37 °C. Substrates were treated with pulsed electric field (PEF) for: 0, 1, 2, 3, 4, 5, 6, 7 or 8 min	Highest biogas and biomethane of 478.0 NmL/g VS and 290.8 NmL CH_4_/g VS were produced from 5 min PEF pre-treatment setups which were 15 and 14 % higher than that of the unpretreated control, respectively	(Szwarc and Glowacka, 2021)
Maize husk	Food waste	Effluent from a reactor with similar substrates	10 L computer-controlled digesters operated at 36 °C for 44 days. The following inoculum/substrate ratios were used: 0, 0.25, 0.5, 1.0, 2.0 and 4.0	Highest peak biogas/methane yields of 0.81/0.52 L/g VS were obtained from 1.0 inoculum/substrate ratio compared to 0.59/0.35 L/g VS from control (without inoculum)	(Owamah et al., 2021)
Food leftovers (FLO)	Human excreta (HE), and kitchen residue (KR)	Anaerobically mono-digested CD	500 mL bottles with 300 mL working volume operated at 30 °C for 61 days	Highest biomethane production of 764.79 mL CH_4_/g VS was produced from the coD of 11.8 % FLO, 78.8 % HE and 9.4 % KR	(Osei-owusu et al., 2024)
Mango peel pulp (MPP)	Municipal mixed sludge (MMS)	–	Digester with 11.3 L working volume operated at 37 °C	Highest biomethane production of 0.47 L CH_4_/g VS was obtained from the coD of 30 % MPP and 70 % MMS compared to 0.26 LCH_4_/g VS produced from the mono-digestion of MMS	(Silva et al., 2024)
Chicken feather	–		500 mL bottles with 300 mL working volume operated at 37 °C and pH of 6.8–7.2	Highest biomethane of 0.67 Nm^3^ CH_4_/kg was produced from chicken feather hydrolysate compared to 0.20 and 0.40 Nm^3^ CH_4_/kg obtained from untreated chicken feather and acetate control, respectively	(Salamony et al., 2024)
Raspberry fruit waste	–	Methanogenic granular sludge from brewery company	Four 4 L reactors with 3 L working volume arrange in parallel, operated at 38 °C and intermittently stirred for 10 min at 60 rpm every 4 h	Average specific biomethane yield of 369 L/kg VS was produced	(Hycienth et al., 2024)
Plant waste: corn or wheat silage; Animal waste: cow or pig manure	–	–	Optimum conditions: residence time of 22 days at either 38 °C or 55 °C	Annual methane production of 1128,262, 295,680, 46,750 and 35,190 Nm^3^ CH_4_/a were obtained from corn silage, wheat silage, cow manure and pig manure, respectively, with an average daily methane value of 4126 Nm^3^/d	(Terziev and Zlateva, 2024)
Poultry manure	–	Anaerobic unit of wastewater treatment plant	Bioreactor of 2 L capacity operated at optimal conditions - 37 °C and pH 6 – 8,	Highest biogas yield of 285 mL/g VS was obtained	(Al-zoubi et al., 2024)
Corn silage	Food waste and digested sludge	Digested sludge	Digester of 20 L volume operated at room temperature, 80 rpm for 30 min (three times daily), optimum HRT of 20 days and total organic loading of 6 g VS/L/d	Highest cumulative biogas of 7279 L/m was produced from the coD with chemically pre-treated corn silage	(Khaita et al., 2024)
Dairy cattle farming waste	–	Anaerobic sludge from agricultural biogas plant	Lab scale: reactor of 1.0 L volume with 0.5 L working volume, operated at 38 °C, 100 rpm (15 min per hour for 24 times) and OLR of ∼5 g/COD/L; Large scale: reactor of 20 m^3^ active volume, operated at 39 °C and OLR of ∼3 kg COD/m	Average biogas and biomethane yields of 367 and 233 mL/g COD, respectively, were obtained at lab conditions while respective biogas and methane yields of 327 and ∼206 mL/g COD were produced at large-scale conditions	(Debowski et al., 2024)
Sugarcane leaves (SCL)	–	Cellulolytic microbial consortium (CMC)	Serum bottle of 120 mL with 70 mL working volume, operated at 37 °C, 7.0 pH and 150 rpm for 45 days	High methane production rate (MPR) of 88.70 mL CH_4_/L.d was produced from pre-hydrolyzed SCL (with CMC) while MPR of 82.57 mL CH_4_/L.d was obtained from SCL augmented with CMC	(Sitthikitpanya et al., 2024)

The proper selection of agricultural waste substrates for coD is essential; otherwise, most of the aforementioned benefits of anaerobic coD might not be achieved. Adequate combinations usually give better biogas and/or biomethane outputs. For example, the coD of vegetable waste plus cow dung was demonstrated to produce more biogas, 0.368 Nm^3^/kg, than that of neem leaf plus vegetable waste, 0.114 Nm^3^/kg (Muhammad and Chandra, 2021). In another study by Meraj et al. (2021) which showed how different combinations of substrates could affect the biomethane outputs of anaerobic coD. There was over 95 % increase in biomethane yield when the substrate combinations was changed from SCB plus rice straw (15.74 NmL CH_4_/g VS) to wheat straw plus rice straw (339.0 NmL CH_4_/g VS) at substrate to inoculum ratios of 2.5 and 1.5, respectively. Therefore, substrate to inoculum ratio is another important parameter to optimize for better biogas/biomethane productions. Other important parameters that should also be optimized, aside basic features like temperature and pH, are co-substrate mixing ratios, organic loading rate (OLR), hydraulic retention time (HRT), etc. (Alrefai et al., 2021; Hortence et al., 2023; Al-zoubi et al., 2024; Sitthikitpanya et al., 2024; Khaita et al., 2024).

Furthermore, the pre-treatment of agricultural waste substrates has been demonstrated to greatly increase both biogas and methane yields in small and large-scale anaerobic biodigesters using mono- or co-substrates. In mono-digestion, higher biogas of 37.8 mL/g and 478.0 NmL/g VS were produced from two different pre-treated agricultural waste substrates – sweet potato root waste (pre-treated with sodium hydroxide) and rapeseed straw (pre-treated with pulsed electric field) – compared to 28.23 mL/g and 406.3 NmL/g VS obtained from the unpretreated samples, respectively. Additionally, there was a respective 22 and 14 % increase in biomethane production in the pre-treated substrates (Catherine and Twizerimana, 2022; Szwarc and Glowacka, 2021). In another study, Sumardiono et al. (2023) demonstrated how different kinds of physical and biological pre-treatments positively impacted on biogas yields from SCB. Higher biogas values of 88.67 and 86.85 mL/g TS were generated from the microbiologically and mechanically (ground) pre-treated substrates compared to 49.12 and 79.9 mL/g TS gotten from the unpretreated substrates, respectively. Similarly, higher cumulative biomethane and biogas of 111.51 mL/g VS and 187.45 L/kg TS were produced from the pre-treated co-cultures of highland barley straw plus cow dung plus pig manure (three substrates) and rice straw plus cow dung (two substrates) than 68.07 mL/g VS and 128.45 L/kg TS obtained from the unpretreated samples, respectively (Jin and Wei, 2023; Sahil et al., 2023).

Adequate sources of seed inocula are also essential in effective biogas production. It is important to use sources that will introduce the needed microbial population required for the different stages of AD. Examples of mostly used inocula sources include: cow dung/manure or fluid from the rumen, sewage sludge, etc. Inoculum could also be obtained from operating anaerobic plants with similar substrates to be digested (Sumardiono et al., 2023; Potdukhe et al., 2021; Sahil et al., 2023; Prajapati and Singh, 2020) (Table 6).

### 2.4 Other biofuels from agricultural waste substrates

Other biofuels can be produced from agricultural wastes. They include biohydrogen, butanol, acetone, etc. Advanced biofuels, i.e. acetone and butanol, can be produced through microbial fermentation via the acetone-butanol-ethanol (ABE) pathway (Valles et al., 2020; Sanguanchaipaiwong and Leksawasdi, 2018). Butanol has a great resemblance to gasoline and it has better features than ethanol when used as fuel. It has a higher heating value and energy content, higher octane number and less corrosivity, as well as lower solubility in water (Sanguanchaipaiwong and Leksawasdi, 2018; Luo et al., 2018; Tri and Kamei, 2020). High biobutanol (≥ 15 g/L) has been produced by wild-type and engineered microbes from different agricultural wastes (Table 7) (Li et al., 2019; Huang et al., 2019; MH et al., 2015).

**Table 7:** Other biofuels production from agricultural wastes.

Biofuel	Agricultural waste substrate	Pre-treatment	Microorganism	Biofuel production	References
Butanol	Apple pomace ultra-filtration sludge (APUtS), suspended brewery liquid waste (SBLW) and starch industry wastewater (SIWW)	Acid hydrolysis (dilute H_2_SO_4_)	*Clostridium beijerinckii*	Optimum biobutanol of 1.4, 1.8 and 4.68 g/L were produced from APUtS, SBLW and SIWW hydrolysates, respectively, compared to the 5.1 g/L obtained from glucose control. Supplementation by 3 % (w/v) glucose increased butanol production to 9.3, 8.06 and 11.04 g/L, respectively	(Maiti et al., 2016)
Butanol	Pineapple waste juice	None	*C. beijerinckii*	A maximum butanol concentration of 3.14 g/L was produced	(Sanguanchaipaiwong and Leksawasdi, 2018)
Butanol	Unbleached hardwood kraft pulp	None	*Phlebia* sp. and *C. saccharoperbutylacetonicum*	Enhanced butanol production of 3.2 g/L from transformed *Phlebia* sp. + *C. saccharoperbutylacetonicum* compared to 2.5 g/L obtained from the parent *Phlebia* sp. + *C. saccharoperbutylacetonicum*	(Tri and Kamei, 2020)
Butanol	Rice straw	Microwave-assisted hydrothermolysis	*C. beijerinckii*	The highest butanol of 5.24 g/L was obtained from simultaneous saccharification and fermentation compared to 4.85 g/L from separate hydrolysis and fermentation	(Valles et al., 2020)
Butanol	Sugarcane molasses and hemicellulose hydrolysate	Acid (dilute H_2_SO_4_)	*C. saccharoperbutylacetonicum*	Butanol yield and titre of 0.31 g g^-1^ and 10 g/L, respectively, were obtained after 72 h of fermentation	(Jiménez et al., 2020)
Butanol	Wheat starch wastewater	None	*C. acetobutylicum*	The highest butanol of 13.51 g/L was produced from the optimized medium supplemented with cassava flour and yeast extract	(Luo et al., 2018)
Butanol	Corncob hydrolysate (CCH)	Acid hydrolysis (dilute H_2_SO_4_)	*Clostridium* sp.	Improved butanol of 4.7 g/L was produced from detoxified (via electrochemical process) pre-treated CCH which was 1.6-fold higher than that obtained from non-detoxified CCH	(Jiang et al., 2021)
Butanol	Sugarcane bagasse (SCB)	–	*C. beijerinckii*	Highest butanol (3.17 g/L) was produced from one-step butanol production	(Phachan et al., 2017)
Butanol	Corn fibre, soyabean hull, cotton stalk and SCB	Acid hydrolysis (dilute H_2_SO_4_ or HCl)	Engineered *C. tyrobutyricum*	Butanol titre and productivity of ∼15 g/L and ∼0.3 g g^-1^, respectively, were obtained from cotton stalk, soyabean hull and SCB	(Li et al., 2019)
Butanol	Pineapple peel waste	Binary acid (phenol and H_2_SO_4_)	*C. acetobutylicum*	Butanol concentration and yield of 5.18 g/L and 0.13 g g^-1^, respectively, were obtained	(Khedkar et al., 2018)
Butanol	Cassava bagasse hydrolysate	Thermal hydrolysis	Engineered *C. tyrobutyricum*	High butanol titre of ˃ 15 g/L and yield of ˃ 0.30 g g^-1^ were obtained	(Huang et al., 2019)
Acetone, butanol and ethanol (ABE)	Sub-standard and surplus date	None	*C. beijerinckii, C. chauvoei* and *C. roseum*	Highest ABE of 24.07 g/L - comprising of 16.16, 7.40 and 0.51 g/L butanol, acetone and ethanol, respectively - was produced by *C. beijerinckii.* While 20.20 and 13.79 g/L were produced by *C. roseum* and *C. chauvoei,* respectively, compared to 11.543 g/L produced by the reference *C. acetobutylicum* ATCC824	(MH et al., 2015)
ABE	Waste starch	None	*C. acetobutylicum*	Highest acetone, butanol and ethanol concentrations of 7.1, 9.9 and 0.4 g/L, respectively, were obtained from 80 g/L initial waste starch	(Kheyrandish et al., 2015)
ABE	Potato peel waste	Several: dilute acid pre-treatment (P1); separate detoxification followed by dilute acid pre-treatment (P2); simultaneous detoxification with organosolv pre-treatment (P3)	*C. acetobutylicum*	ABE concentration of 11.6 g/L was produced from P2 while enzymatic hydrolysis of substrates from P2 gave highest ABE of 24.8 g/L	(Abedini et al., 2020)
ABE	*Amorphophallus* konjac waste	None	*C. acetobutylicum*	Highest ABE solvents of 10.95 g/L were produced from separate hydrolysis and fermentation while simultaneous saccharification and fermentation gave 6.64 g/L ABE compared to 4.34 g/L from the control (with glucose/mannose)	(Shao and Chen, 2015)
ABE	Corn stover hydrolysate (CSH)	Steam explosion	*C. acetobutylicum*	High ABE titer of 17.68 and 17.05 g/L were produced from undetoxified pretreated-SCH in 1 L and 1 m^3^ bioreactors, respectively	(Su et al., 2024)
Biobutanol and ABE	Cranberry bush fruit pomace	Acid pre-treatment and enzymatic hydrolysis	*C. beijerinckii*	A maximum biobutanol and total ABE of 9.45 and 12.08 g/L were produced by 20 % inoculum at 72 h	(Nur et al., 2024)

Several species of *Clostridium* such as *C. beijerinckii, C. saccharoperbutylacetonicum, C. acetobutylicum, C. roseum,* etc. have been demonstrated to be capable of producing butanol and ABE from agro-forestry waste substrates (Table 7). Usually, these lignocellulose biomass are pre-treated chemically (with acid or base), thermally, electrochemically or enzymatically before fermentation (Jiménez et al., 2020; Huang et al., 2019; Jiang et al., 2021; Abedini et al., 2020; Khedkar et al., 2018). Jiang et al. (2021) reported that such pre-treatment and removal of the resultant inhibitors by electrochemical process helped to enhance butanol production. They recorded about 1.6-fold increase in butanol production after the detoxification pre-treatment. Furthermore, saccharification of the complex carbohydrate could be done separately before fermentation (SHF) or simultaneously with fermentation (SSF). Several researchers have reported varying impacts of SHF and SSF on biobutanol yield (Table 7) (Valles et al., 2020; Shao and Chen, 2015).

## 3 Remediation and/or valorization of biofuel agricultural waste substrates and by-products

The application of agricultural wastes as substrates for biofuel production is such an interesting concept that has future potential to making biofuels competitive (if not cheaper) to their fossil fuel counterparts. Ordinarily, these wastes are usually sources of menace in the environment when improperly disposed of. On the other hand, the adequate treatment of these wastes could be a huge financial burden considering the annual volume of agricultural wastes being generated globally (∼998 million tons) (Raut et al., 2023; Siddiqua et al., 2022; Abubakar et al., 2022).

**Fig. 2:**
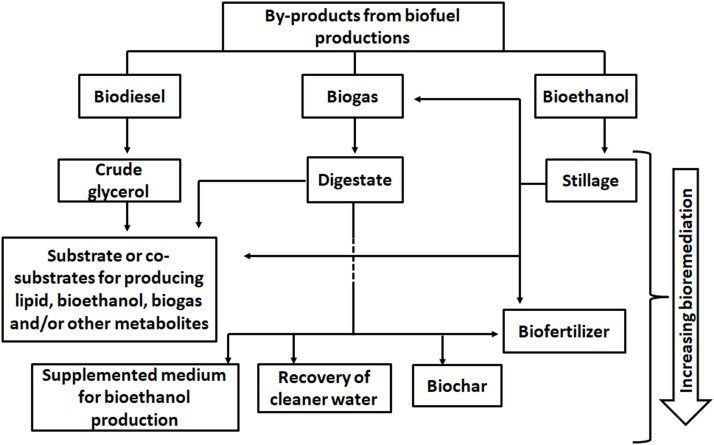
Valorization of by-products from microbial biofuel productions.

The valorization of these wastes into different biofuels while simultaneously remediating them is a win-win option. This channel encourages a global zero-waste and circular bioeconomy (Vyas and Chhabra, 2019; Karim et al., 2021; Venkatesh, 2022; Sarangi et al., 2023). Furthermore, the by-products from agricultural waste substrates that were used for biofuels are also been valorized. For example, the crude glycerol from biodiesel generation has been demonstrated in several works to be an alternative carbon source to grow microorganisms (Liu et al., 2017; Yen et al., 2019; Louhasakul et al., 2020). The stillages from bioethanol production are been explored in biogas production and other applications (Wang et al., 2014; Liu et al., 2015; Kotarska et al., 2019). While the by-products from biogas are employed as rich sources of biofertilizers and biochar (Jin et al., 2022; Hung et al., 2017; Ndubuisi-Nnaji et al., 2020; Casini et al., 2021). Recent research works have shown that the anaerobic digestate (i.e. waste effluents) from biogas production can further be valorized to generate bioethanol, lipids for biodiesel, fumaric acid, etc. These are coupled with the recovery of cleaner water and heavy metal remediation (Fig. 2) (Koutra et al., 2019; Ujor et al., 2020; Agyeman-Duah et al., 2023).

### 3.1 Simultaneous remediation of agricultural waste substrates used for biofuels

The other interesting benefit of using agricultural wastes in microbial biofuel production is the simultaneous bioremediation of these waste substrates which would have cost a lot in treatment before proper disposal or constitute environmental pollution without due treatment. Using these wastes as substrates therefore adds great value to them (Fig. 2). Several researchers have reported over 90 % remediation of some agricultural wastes – in terms of total nitrogen, total phosphorus, total solids, biochemical oxygen demand, or chemical oxygen demand removal – during fermentation. These wastes were used as sole or co-substrates (Table 8) (De Bhowmick et al., 2019; Nurjuwita et al., 2021; Okpozu et al., 2019; Patel et al., 2020; Marín et al., 2022).

**Table 8:** Simultaneous biofuel production and bioremediation of substrates.

Agricultural waste	Microorganism	Biofuel/lipid/carbohydrate production	Bioremediation	Reference
Poultry litter	Microalga: *Acutodesmus obliquus*	Highest lipid of 38.49 mg/L was obtained from pre-treated poultry litter extract (PPLE)	Higher NH_3_-N, NO_3_-N and PO_4_-P removal of 81.82, 79.51 and 80.52 %, respectively, were achieved using PPLE	(Musetsho et al., 2021)
Cassava wastewater	Microalga: *Desmodesmus* *armatus*	Highest lipid content of 21.91 % was obtained in the mixotrophic culture condition	Highest reduction in total dissolved oxygen, electricity conductivity, biochemical oxygen demand (BOD) and chemical oxygen demand (COD) by 83, 83, 87 and 92 %, respectively, in mixotrophic culture condition	(Okpozu et al., 2019)
Kitchen waste and poultry litter	Microalga: *Chlorella* *minutissima*	Highest lipid content of 58 % was obtained	100 % removal of nitrate, ammonia and nitrite with 85 and 91 % removal of carbon and phosphorus, respectively	(De Bhowmick et al., 2019)
Swine wastewater	Microalga: *Parachlorella kessleri*	Carbohydrate (CHO) productivity of 646 mg/L.d	COD, total nitrogen (TN) and total phosphate (TP) removal of 88, 95 and ∼100%, respectively	(Qu et al., 2019)
Fish silage	Microalga: *Scenedesmus* sp.	Enhanced CHO and lipid of 44.90 and 84.21 mg/d DW, respectively, were produced	∼90 % effective reduction in nitrate, ammonia and phosphorus	(Abdulsamad and Varghese, 2017)
Food waste	Microalga: *Scenedesmus obliquus*	Highest CHO and lipid productivities of 13–16 and 10–11 mg/L/d, respectively, were obtained from food waste plus flue gas CO_2_	Nutrient removal of 21–22 mg TN/L was achieved in mixotrophic condition	(Ji et al., 2015)
Whey	Microalga: *Chlorella protothecoides*	Highest lipid of 1.90 g/L with daily productivity of 0.20 g/L/d were obtained	Removal of 91–100 and 99.7 % of inorganic and organic pollutants, respectively	(Patel et al., 2020)
Cheese whey (CW)	Yeast: *Cystobasidium* *oligophagum*	Highest lipid content and productivity of 44.12 % and 0.0335 g/L h, respectively, were obtained from deproteinized CW	Soluble COD removal rates of 8.049 and 10.610 g/L day were obtained for deproteinized CW and untreated CW, respectively	(Vyas and Chhabra, 2019)
POME	Yeast (*Lipomyces starkeyi*) and bacterium (*Bacillus cereus*) co-culture	Highest lipid production of 2.27 g/L was obtained	Maximum COD removal of 83.66 % was achieved	(Karim et al., 2021)
Tofu liquid waste and cow dung	Effective microorganisms 4	Highest biomethane content of 90.351 % was obtained	Highest COD, BOD and total soluble solids removal of 91.12, 97.52 and 91.22 %, respectively, were achieved	(Nurjuwita et al., 2021)
Food waste	Dominant algae: *Pseudoanabaena* sp. and *Chlorella vulgaris*	An average biogas of 790 mL/g VS was produced	100 % removal of both TN and TP	(Marín et al., 2022)
	–	Best biomethane and biohydrogen of 381.83 and 154.91 mL/g VS were produced	COD of 72.97 - 82.86 % removal was achieved	(Gu et al., 2024)
Chicken feather	*Bacillus subtilis* (recombinant strain)	Maximum biomethane and biohydrogen of 0.67 Nm^3^ CH_4_/kg and 0.85 mmol H_2_/day.L were produced	COD removal of 86 and 93 % were achieved in the generation of biomethane and biohydrogen, respectively	(Salamony et al., 2024)
Rice straw	–	Highest total cumulative biogas production of 15.03 L was obtained	Highest COD removal efficiency of 70 % was achieved	(Ahmed et al., 2024)

Microalgae, yeasts and bacteria have been applied in the remediation of several agricultural wastes like poultry litter, cassava and swine wastewater, POME, food waste, whey, etc. Examples of these microorganisms include: several species of *Chlorella* and *Scenedesmus, Desmodesmus armatus, Lipomyces starkeyi, Cystobasidium oligophagum, Bacillus* sp. etc. These oleaginous microbes accumulated good amount of lipids while biodegrading the substrates simultaneously (Table 8). Also, in the production of biogas, several agricultural waste substrates, such as tofu wastewater, cow dung, food waste, etc., have been reported to be adequately remediated with ample biomethane generation. Marín et al. (2022) achieved a 100 % removal of total nitrogen and phosphate during biogas production from food wastes using a 100 L anaerobic digester that was integrated into an outdoor high-rate microalgal pond of 1.2 m^2^ capacity.

### 3.2 Valorization of biofuel by-products from agricultural wastes

One major application of biofuel by-products, especially biogas digestate, is in the production of biofertilizers. Biofertilizer is one of the major cost-effective components of an integrated nutrient management system that acts as a renewable source of plant nutrients due to the presence of microbes for a more sustainable agriculture which has a lot of benefits above chemical fertilizers (Ahamefule et al., 2024; Kumar et al., 2016). Achieving zero-waste in the biorefinery system can be made possible with the use of sustainable agricultural waste substrates while also utilizing the by-products from the waste substrates as useful resources which leads to a circular bioeconomy (Sarangi et al., 2023).

By-products obtained from biorefinery system require further processing for other synthesis as opposed to a direct use of resources, thereby minimizing the waste generated (Ubando et al., 2020). Gabra et al. (2019) showed that the waste fermentation culture of N_2_-fixing *Bacillus* sp. that was reused produced biofertilizer, thereby limiting the process of disposal or inactivation either by chemical treatment or heat. The economic feasibility of biofuel production can be increased by reusing the bacterial biomass as biofertilizer, thus reducing the cost of biofuel production as well as the economic track of synthetic fertilizers (Gabra et al., 2019). According to Kefalew and Lami (2021), biogas produced from abattoir was able to generate a large yield biofertilizer which could amount to 43,184.9 kg per year while lowering greenhouse gas emissions. Furthermore, the biofertilizer produced from the by-products of biogas has the capability of raising the yield of crops from 15 % to 25 % when applied.

The key to utilizing currently disposed agricultural by-products might be the production of biochar as fuel or soil amendment. Biochar, according to the International Biochar Initiative, is a solid carbonaceous residue obtained in the absence of oxygen at a controlled temperature after the thermochemical degradation of several biomass feedstocks (Pourhashem et al., 2019). It can be applied as an absorbent, a catalyst, agent of soil amendment and for other nonfuel uses (Awogbemi and Von, 2023). Pyrolysis is used for the production of biochar in an oxygen deficient system by devolatilizing the biomass to get high quality fuel. Biochar obtained via pyrolysis is considered more desirable as a result of the various applications as well as the energy-generating potential and low carbon footprint (Ennis et al., 2012; Oh et al., 2021; Ganesapillai et al., 2023). Some studies have shown that biochar could be produced using biogas by-products (Hung et al., 2017; Casini et al., 2021).

Heat and electricity generated from burning biomass residue are expected to provide the energy required for processing biomass in an ideal biorefinery plant, which ensures economic feasibility (Cherubini, 2010). Renewable electricity can be produced by biochar, which is made possible by the combustion of feedstock to generate steam. And for the generation of electricity in coal-firing plants, the substitution of coal with biochar as feedstock has great potential for the reduction of carbon dioxide emissions globally (Osman et al., 2023).

## 4 Challenges and prospects in the valorization of agricultural wastes into biofuels

As interesting as the valorization of agricultural wastes into biofuels may be, there are still some inherent challenges that limit the process. A bulk of agro-forestry wastes consist of lignocellulose biomass. Lignocellulose agricultural crop residues are composed of cellulose (23 – 32 %), hemicellulose (38 – 50 %) and lignin (10 – 25 %) (Meraj et al., 2021). The recalcitrant lignin structure usually prevents access to the cellulose and hemicellulose components (Zoghlami and Paës, 2019; Wu et al., 2023; Jönsson and Martín, 2016). Several kinds of pre-treatments – ranging from physical to enzymatic - are applied to remove the lignin layers (Valles et al., 2020; Khedkar et al., 2018; Maiti et al., 2016). Unfortunately, some inhibitory substances such as furfural, acetic and formic acids, etc., generated by pre-treatments do prevent and/or limit microbial growth if they are not properly removed from the pre-treated substrates (Jönsson and Martín, 2016; Ndubuisi et al., 2020). The whole process of pre-treatment and removal of these inhibitory substances could add to the cost of production, especially in large-scale systems. However, the isolation of more microorganisms that could still use the substrate without pre-treatment and/or that have high tolerance to these inhibitory substances would go a long way to addressing this challenge. Genetically engineering potential microorganisms to do these is yet another viable option.

After pre-treatment, there is a need to saccharify the released complex carbohydrates (i.e. cellulose and hemicellulose) to simple sugars that can easily be utilized by microorganisms. Enzymatic hydrolysis has been used in several studies (Sharma and Sharma, 2023; Mihajlovski et al., 2021; Sanjivkumar et al., 2018). However, the cost of commercial enzymes could increase the production cost of the process. Therefore, obtaining microorganisms (either individual isolates or consortia) that can produce these enzymes is very essential. Harvested crude enzymes from such microbes could be utilized directly or the microorganisms could be co-cultured with the biofuel-producing organisms as in the cases of either SHF or SSF in bioethanol or biobutanol productions (Sharma and Sharma, 2023; Srivastava et al., 2021; Yan et al., 2018; Mihajlovski et al., 2021; Pandey et al., 2020). The search for consolidated biofuel-producing microorganisms has greatly reduced the cost and cumbersome nature of these whole processes. For example, one microorganism can now saccharify and ferment or produce lipids (Sanjivkumar et al., 2018; Buraimoh et al., 2021; Nisha et al., 2017; Carrillo-Nieves et al., 2022). To further address this challenge, very good oleaginous or bioethanol-producing microbes could be transformed to express lignocellulase enzymes for hydrolysis.

Another compelling challenge of using agricultural wastes for biofuel production is the possibility of variations in the quality and/or quantity of biofuel production due to the seasonality of agricultural products. Since most crops, fruits and vegetables are seasonal, the wastes generated from them will also be seasonal. Furthermore, variation of substrates (due to availability) may also pose a big challenge to microorganisms used in the fermentations for biofuels (Zhao et al., 2022; Bonuedi et al., 2022). Although the seasonality of these substrates might be challenging to address directly until the achievement of major food/fruits/vegetable productions all year round, however, some other factors could be considered to ameliorate this problem. The development of adequate starter cultures and consortia, including novel isolates and transformed microorganisms, that could hydrolyze and metabolize any/several kind(s) of agricultural wastes (based on their compositions) could be a huge step forward to addressing this challenge. As such microbes could metabolize any agricultural waste; there would be no serious problem with different wastes generated with seasons. More so, the characterization of major agricultural waste substrates used in biofuel production could help in moderating and obtaining the right constitutes and proportions needed for fermentation when using other available waste products.

Most of these biofuel plants are not located in the farms and city hubs where these agricultural wastes are generated, the logistics of packaging and transporting these substrates to the plants might be a big challenge. This challenge is currently been addressed by integrating biofuel production units into some major farms like sugarcane plantations as obtainable in Brazil. Therefore, the vinasse, stillage and molasses wastes generated from sugarcane would be transferred to the biofuel units where they are used for bioethanol, biobutanol, biogas, etc. (Xu et al., 2017; Volpi et al., 2022; Herman et al., 2022). Integrated biofuel production could be the most interesting avenue to addressing this challenge as different processes (including biofuels and other essential products) would be designed and coupled into a biorefinery to prevent the cost of moving one by-product (which is a substrates for another process) far away (Ahamefule et al., 2024; Rabie, 2022; Lin et al., 2017; Kanlayavattanakul and Lourith, 2023). However, the technicality and cost of designing and operating such holistic biorefinery is yet another issue that is only gradually been addressed globally.

## 5 Conclusions

The United Nations estimates that global population will reach 9.7 billion by 2050. The rise in population will attract an increase in energy and food demands. The continual use of fossil fuels with such an increase in population rise would escalate the current global climate change issues. Additionally, success in the production of food that would cater for such a population would also lead to more agricultural waste which is currently estimated at 998 million tons annually. Developing and expanding technologies and refineries that would convert these wastes into biofuels via microbial processes is the only cost-effective method to produce green energy while also managing and/or eradicating waste pollution.

## CRediT authorship contribution statement

**Chukwuemeka Samson Ahamefule:** Conceptualization, Writing – original draft, Writing – review & editing. **Chidimma Osilo:** Writing – review & editing. **Blessing C. Ahamefule:** Writing – review & editing. **Stella N. Madueke:** Writing – review & editing. **Anene N. Moneke:** Writing – review & editing, Supervision.

## Declaration of competing interest

The authors declare that they have no conflict of interest.

## Data Availability

No data was used for the research described in the article.
